# Gene expression profiles separate endometriosis lesion subtypes and indicate a sensitivity of endometrioma to estrogen suppressive treatments through elevated *ESR2* expression

**DOI:** 10.1186/s12916-023-03166-1

**Published:** 2023-11-23

**Authors:** Sushma Marla, Sally Mortlock, Taija Heinosalo, Matti Poutanen, Grant W. Montgomery, Brett David McKinnon

**Affiliations:** 1 Institute for Molecular Bioscience, The University of Queensland, Carmody Rd, Brisbane, QLD 4067 Australia; 2 Institute of Biomedicine, Research Centre for Integrative Physiology and Pharmacology, University of Turku, Turku, 20014 Finland; 3 Turku Center for Disease Modelling, University of Turku, 20014 Turku, Finland

**Keywords:** Endometriosis, Oestrogen receptor, Treatment, Heterogeneity, Subtypes, Gene expression

## Abstract

**Background:**

Endometriosis is a common, gynaecological disease characterised by the presence of endometrial-like cells growing outside the uterus. Lesions appear at multiple locations, present with variation in appearance, size and depth of invasion. Despite hormones being the recommended first-line treatment, their efficacy, success and side effects vary widely amongst study populations. Current, hormonal medication for endometriosis is designed to suppress systemic oestrogen. Whether these hormones can influence the lesions themselves is not yet clear. Evidence of hormone receptor expression in endometriotic lesions and their ability to respond is conflicting. A variation in their expression, activation of transcriptional co-regulators and the potential to respond may contribute to their variation in patient outcomes. Identifying patients who would benefit from hormonal treatments remain an important goal in endometriosis research.

**Methods:**

Using gene expression data from endometriosis lesions including endometrioma (OMA, *n* = 28), superficial peritoneal lesions (SUP, *n* = 72) and deeply infiltrating lesions (DIE, *n* = 78), we performed principal component analysis, differential gene expression and gene correlation analyses to assess the impact of menstrual stage, lesion subtype and hormonal treatment on the gene expression.

**Results:**

The gene expression profiles did not vary based on menstrual stage, but could distinguish lesion subtypes with OMA significantly differentiating from both SUP and DIE. Additionally, the effect of oestrogen suppression medication altered the gene expression profile in OMA, while such effect was not observed in SUP or DIE. Analysis of the target receptors for hormonal medication indicated *ESR2* was differentially expressed in OMA and that genes that correlated with *ESR2* varied significantly between medicated and non-medicated OMA samples.

**Conclusions:**

Our results demonstrate of the different lesion types OMA present with strongest response to hormonal treatment directly through *ESR2*. The data suggests that there may be the potential to target treatment options to individual patients based on pre-surgical diagnoses.

**Supplementary Information:**

The online version contains supplementary material available at 10.1186/s12916-023-03166-1.

## Background

Endometriosis is an oestrogen-dependent gynaecological disease associated with chronic pelvic pain and infertility and is characterised by the growth of endometrial tissue outside the uterine cavity. It is a heterogeneous disease both in phenotype and clinical outcome (1). Current treatment is either via oestrogen suppression or the surgical removal of lesions. Surgical removal can be difficult and associated with complications. Lesions will also reoccur in up to 20% of patients within 3 years [1] and 50% of patients within 5 years [2]. Oestrogen suppression is achieved via hormonal preparations that suppress systemic production, which can be accompanied by unwanted side effects [3].

Endometriosis lesions are found throughout the peritoneal cavity. They are significantly heterogeneous with variation in size, colour, appearance, location and morphology [4]. The association between appearance, clinical symptoms and response to treatment remain unclear. Currently, lesions are categorised into superficial peritoneal (SUP), ovarian endometrioma (OMA) and deeply infiltrating endometriosis (DIE), often considered the most severe form of the disease [5] and characterised by penetration of greater than 5 mm into the underlying tissue. Whether these lesions represent distinct subtypes [4] or a continuum of disease progression is not yet clear [6]. Gene expression differences between the three lesion subtypes have been reported [7].

Estrogen suppression in endometriosis patients reduces symptoms [8, 9] and inhibits lesion growth [9], and post-surgical hormonal treatment can reduce recurrence [10]. Many women, however, show no response, or prohibitive side effects and a trial-and-error approach to various hormonal preparations is often applied to find an acceptable treatment [11]. Why some hormonal preparations are effective for some women, but not others is not clear. The proteins targeted by these hormones, including progesterone (PGR) and oestrogen receptors (ER), have been shown in endometriosis lesions [12, 13] and their expression is influenced by the microenvironment [14, 15] and associated with treatment response [16, 17]. Local interactions between treatments and lesions could contribute to individual differences to the response.

Using genome-wide gene expression data from multiple deeply phenotyped datasets [7, 18] and advanced bioinformatic analysis, we investigated whether there were fundamental differences between menstrual cycle stage, known subtypes, and the influence of hormonal treatment on endometriotic lesion gene expression. Assessment of critical genes and co-regulators that mediate treatment response were further analysed.

## Methods

### Gene expression in endometrium and endometriosis

#### Dataset A

**Fig. 1:**
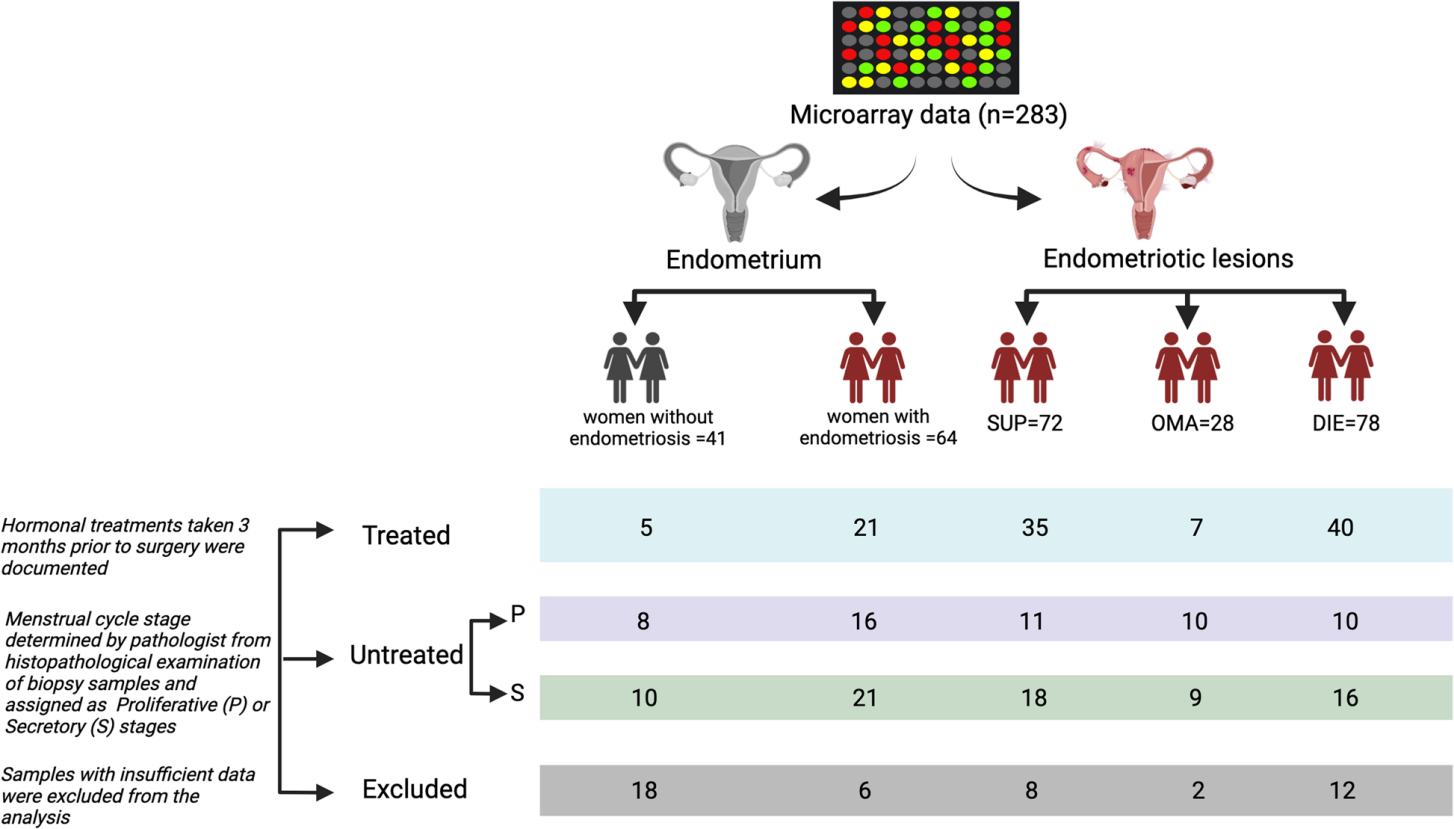
Description of the clinical characteristics of endometrium and endometriotic lesions samples from Dataset A. Dataset A contains the gene expression data from a total of 283 samples of endometrium (*n* = 105) and endometriosis lesions (*n* = 178). The number from each menstrual cycle phase and with and without treatment is also shown

Gene expression and accompanying phenotypical data was provided through collaboration with the University of Turku with the gene expression data available from the Gene expression omnibus (GEO) GSE141549 [19, 20]. This data included gene expression for 283 samples from the endometrium of patients with (*n* = 64) or without (*n* = 41) endometriosis and endometriosis lesions (*n* = 178). All samples were hybridised to Illumina Human 6 V2 arrays containing 48,701 probes. Endometriosis lesions were classified as either SUP (*n* = 72), OMA (*n* = 28) or DIE (*n* = 78). Hormonal treatments taken within 3 months prior to surgery were documented during clinical examination and used to assign samples treated or untreated (Additional file 1: Table S1). Menstrual cycle stage was determined by an experienced pathologist from histopathological examination of endometrial biopsy samples [21] (Fig. 1).

#### Dataset B

A second, previously published, dataset was used to evaluate endometrial gene expression profiles [18]. Briefly, the dataset contained 229 endometrial tissue samples from patients of European ancestry attending clinics at the Royal Women’s Hospital or Melbourne IVF in Melbourne, Australia with (*n* = 161) and without (*n* = 67) endometriosis. Expression data were generated on the Illumina Human HT-12 v4.0 bead chip microarray. Histologic evaluation classified these samples into Menstrual (M) = 11, Proliferative (P) = 94 and Secretory (S) = 124 stages.

To evaluate the consistency of gene expression across datasets, we used all endometrial samples to perform a correlation analysis between the gene expression in dataset A and dataset B. A subsequent correlation was also determined after splitting the samples between the proliferative and secretory stages.

### Gene expression normalisation

Normalisation techniques for gene expression in both datasets were performed as previously described [18, 22]. Data were pre-processed using Illumina GenomeStudio software (Illumina Inc., San Diego) where data was background corrected and any probes with a detection *p*-value less than 0.05 was considered expressed in that sample. Data was subsequently quantile normalised and log transformed. In total, 48,701 probes were expressed in dataset A and 47,235 probes were expressed in dataset B.

### Factors contributing to variation in gene expression

Principal component analysis (PCA) was used to evaluate variation in gene expression between lesion samples in the dataset A. PCA was performed on normalised log-transformed expression values using the prcomp function in R (V4.1.3). ANOVA was used to test the association between the top five PCs as well as the menstrual cycle stage and lesion subtype (SUP, OMA, DIE).

### Identifying differentially expressed genes (DEGs)

To avoid introducing expression bias with genes not expressed in some samples, the analysis was restricted to genes expressed in > 90% of all samples, leaving 14,747 probes in dataset A and 12,247 probes in dataset B [18].

In endometrial samples, differential gene expression was performed using the eBayes method in the limma package (R version-4.1.3). A comparison was conducted between the proliferative and secretory stages with endometriosis status as a covariate in each dataset. Within dataset A differential expression between samples collected from those taking and not taking hormonal medication was also tested with menstrual stage as a covariate. The Pearson correlation coefficient was used to determine the correlation between log-transformed gene expression in the two datasets.

In endometriotic lesions, normalised and batch-corrected gene expression levels were used to conduct differential gene expression analysis between samples collected at different menstrual cycle stages. This was conducted in lesions of all subtypes, as well as in each subtype separately (SUP v DIE; SUP v OMA; DIE v OMA). *P*-values were corrected for multiple testing using the Bonferroni method and Benjamini–Hochberg method and an adjusted *p*-value less than 0.05 was considered statistically significant. The top 50 differentially expressed genes (DEGs) were visualised and clustered using the heatmap function in R.

### Expression and correlation of hormone receptors

Using the normalised gene expression dataset, gene expression across lesion subtypes for nine hormone receptors (oestrogen receptor 1 (*ESR1*), oestrogen receptor 2 (*ESR2*), androgen receptor (*AR*), *PGR*, progesterone receptor membrane component 1 (*PGRMC1*), progesterone receptor membrane component 2 (*PGRMC2*), gonadotropin-releasing hormone 1 (*GnRH1*), gonadotropin-releasing hormone 2 (*GnRH2*), mineralocorticoid receptor (*MCR*)) was investigated using a two-way ANOVA. Some genes were not expressed in more than 90% of samples; hence, this analysis was not restricted. Covariates included were batch number, menstrual stage and medicated status. A *p*-value of 0.05 or less was deemed statistically significant.

A genome-wide correlation analysis was conducted on the medicated and un-medicated samples separately for *ESR1* across all lesion subtypes and for *ESR2* in OMA subtype only. Pair-wise correlations were performed controlling for the false discovery rate (FDR) using the Benjamini–Hochberg method. An FDR < 0.05 was considered significant.

### Cell type enrichment analysis

Using the xCell pipeline in R [23], cell-type enrichment analysis was performed on dataset A to estimate the cell-type composition in each sample from lesion data. xCell is a bioinformatics application that generates cell type enrichment scores by comparing expression profiles to reference data for 64 cell types.

### Biological pathway analysis

ClusterProfiler [24], which is capable of analysing and visualising data for enrichment analysis, was utilised to acquire better biological insight into differentially expressed genes identified as significant from the differential gene expression analysis. Enrichment was considered significant if Benjamini–Hochberg adjusted *p*-values were less than 0.05.

## Results

### Consistency of gene expression across independent datasets

**Fig. 2:**
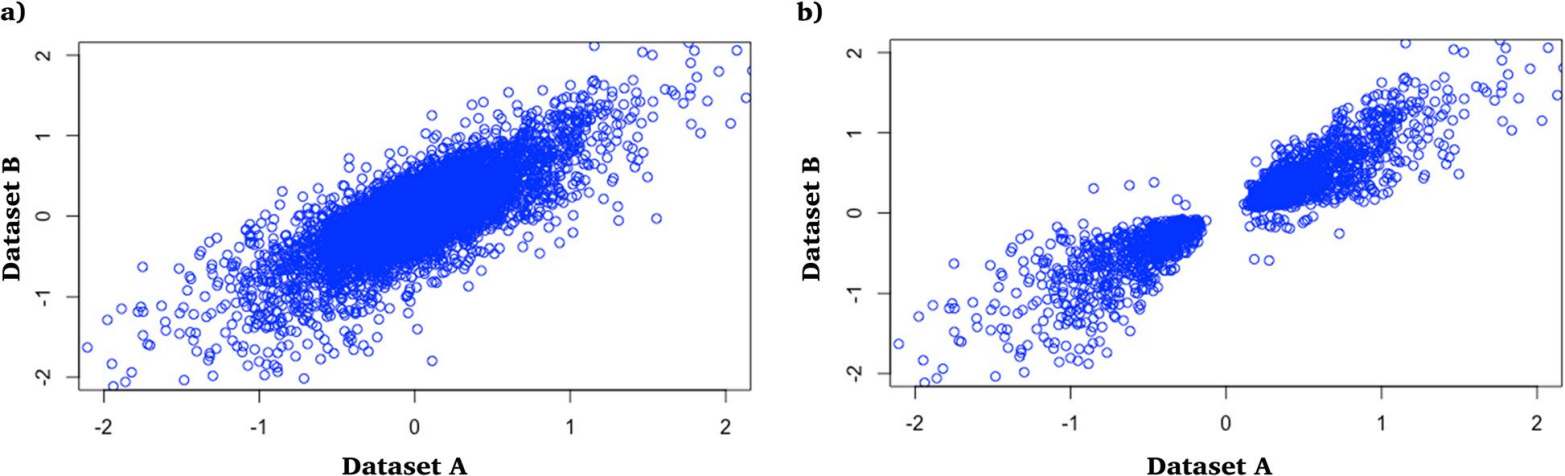
Gene expression correlation of Finnish and Australian datasets. **a** Gene expression in the Finnish (Dataset A; *n* = 55) and Australian (Dataset B; *n* = 212) dataset showed a significant correlation (*r*^2^ = 0.7) of all non-medicated endometrial samples. **b** Stratification via menstrual stage and selection for genes with significantly different expression between menstrual cycle stages also showed a significant correlation (*r*^2^ = 0.9) between the two datasets

The correlation between expressed genes was assessed to establish consistency across independent datasets. A direct comparison of constitutively expressed endometrial genes in the endometrial samples from all patients revealed a significant correlation between dataset A and dataset B (*r*^2^ = 0.70) (Fig. 2a). Comparing the gene expression in the endometrial samples from the proliferative and secretory stages independently identified 3202 and 8361 FDR significant DEG in dataset A and dataset B, respectively. The correlation of these DEG between the two datasets was also significant (*r*^2^ = 0.9) (Fig. 2b), indicating the consistency of endometrial gene expression across datasets generated at different locations, using different microarray chips, and from individuals of different European ancestry.

### Influence of hormonal medication on endometrium gene expression

**Fig. 3:**
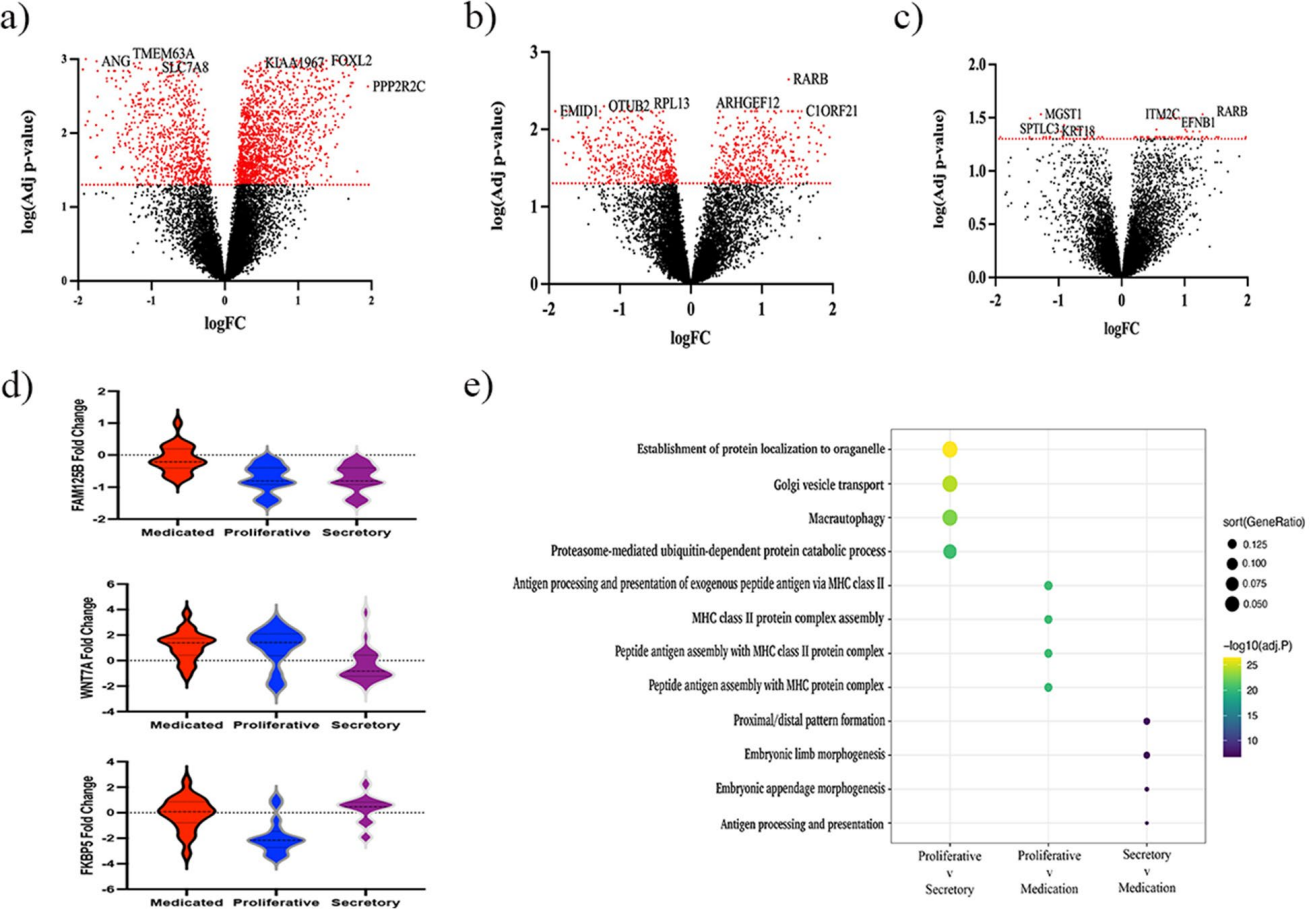
Influence of hormonal treatment on endometrial gene expression. Volcano plots showing **a** differential gene expression between the proliferative and secretory stage identified 1791 genes that were significantly upregulated and 1044 genes that were significantly downregulated. A comparison of the medicated samples compared to the **b** proliferative stage and **c** the secretory stage found only 1102 and 77 genes differentially regulated respectively. **d** Violin plots showing the fold change of genes influenced by medication including *FAM125B*, *WNT7A* and *FKBP5* that showed similar regulation between medicated and secretory samples. **e** Pathway analysis using the gene set enrichment analysis (GSEA) identified medication influenced immune system pathways in endometrium tissue. The top four pathways for each comparison are shown in the dotplot. The size of the dot is relative to ratio of gene enrichment and the colour shows the significance of the enrichment as a − log10 adjusted *p*-value

As endometriosis status may have a confounding effect on endometrial gene expression [18], analysis was limited to cases exclusively. A comparison of endometrial samples from the proliferative (*n* = 16) and secretory (*n* = 21) stages revealed 2835 DEGs following FDR correction and 142 DEGs following Bonferroni correction (Additional file 1: Table S2; Fig. 3a), confirming the influence of cycle stage in cases only. A pair-wise comparison of hormone-medicated (*n* = 21) endometrium samples and endometrium from the un-medicated proliferative stage identified 1012 DEGs following FDR correction and six DEGs following Bonferroni correction (Additional file 1: Table S3; Fig. 3b), and with endometrium from the un-medicated secretory stage samples identified 77 DEGs following FDR correction (Additional file 1: Table S4; Fig. 3c). No genes were significantly different between hormone-medicated samples and the secretory stage following Bonferroni correction.

Of the 1012 DEG in the medicated and proliferative stage endometrial samples, a total of 562 genes were upregulated and 450 were downregulated in medicated samples. In contrast, 32 were upregulated in medicated samples and 45 downregulated compared to the secretory stage. The influence of the menstrual stage and hormones created distinct profiles for each gene. *FAM125B* was significantly upregulated in medicated compared to secretory stage samples (*p*_prol_ = 0.10; *p*_secr_ = 2.58 × 10^−5^). *WNT7A* displayed similar concentrations in medicated and proliferative stage samples but was significantly upregulated compared to secretory stage samples (*p*_prol_ = 0.77; *p*_secr_ = 0.00024). *FKBP5* expression was similar between medicated and secretory stage samples, but significantly upregulated compared to proliferative stage samples (*p*_prol_ = 9.51 × 10^−5^; *p*_secr_ = 0.26) (Fig. 3d). A gene ontology analysis suggested that the major distinction between proliferative and secretory stages is cellular processes (Fig. 3e). In contrast, medicated samples demonstrated change in MHC class II protein activity relative to the proliferative stage and morphogenesis variation compared to the secretory stage. Combined together this data confirms hormonal treatment significantly influenced gene expression in endometrium. It influenced each gene differently, although the gene expression profiles of medicated samples appear more similar to the secretory stage, as evidenced by the smaller number of DEGs, compared to the proliferative endometrium.

### Endometriotic lesion gene expression is influenced by subtype not menstrual stage

**Fig. 4:**
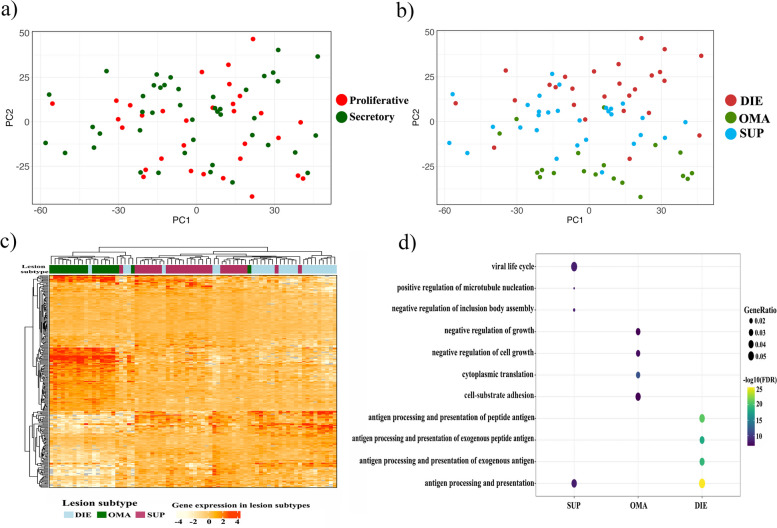
Gene expression profiles of endometrial lesions. **a** PCA of gene expression variability in all endometriotic lesions coloured by menstrual cycle phase at sample collection. No association between gene expression and the menstrual stage was observed. **b** PCA analysis of all samples coloured by lesion subtype. A significant separation by lesion subtype was observed. **c** Heatmap of genes differentially expressed between lesion subtypes. Comparison of gene expression clearly indicated samples clustered predominantly based on lesion subtype. List of genes used for sample clustering is available in Tables S4, S5 and S6. **d** Analysis of biological pathways enriched for genes differentially expressed between different subtypes (SUP, OMA, DIE). Samples from DIE displayed an increased response to antigens, whereas OMA showed a negative regulation of growth

To explore gene expression differences in lesions, we first assessed the menstrual cycle given its significant influence on the endometrium. In contrast to the endometrium, no significant association was observed between variation in gene expression and different cycle stages in the lesions (Fig. 4a). Of these lesions, 29 were SUP (11 proliferative and 18 secretory), 19 were OMA (10 proliferative and 9 secretory), and 26 were DIE (10 proliferative and 16 secretory). In lesions stratified by subtype, the influence of menstrual stage on gene expression for SUP, OMA, or DIE was similarly not significant.

PCA revealed clustering of samples with PC2 significantly associated with subtype (*p* = 1.37 × 10^−13^) suggesting subtype could explain a significant amount of variability in gene expression (Fig. 4b). Analyses of differential gene expression between SUP and DIE found 1108 and 27 DEGs following FDR and Bonferroni correction, respectively (Additional file 1: Table S5). Differential expression analysis between SUP and OMA found 1677 and 202 DEGs following FDR and Bonferroni correction, respectively (Additional file 1: Table S6). Between OMA and DIE lesions; 2663 and 334 DEGs following FDR and Bonferroni correction, respectively (Additional file 1: Table S7). A heatmap analysis based on top 50 genes from each combination confirmed OMA clustered separately from the DIE and SUP lesions. Additionally, the DIE and SUP lesions also formed separate clusters (Fig. 4c). Gene ontology analysis revealed that differences in gene expression drove variation in pathways relevant to antigen processing presentation in peritoneal lesions, while DIE lesions showed variation in growth regulation pathways (Fig. 4d).

**Fig. 5:**
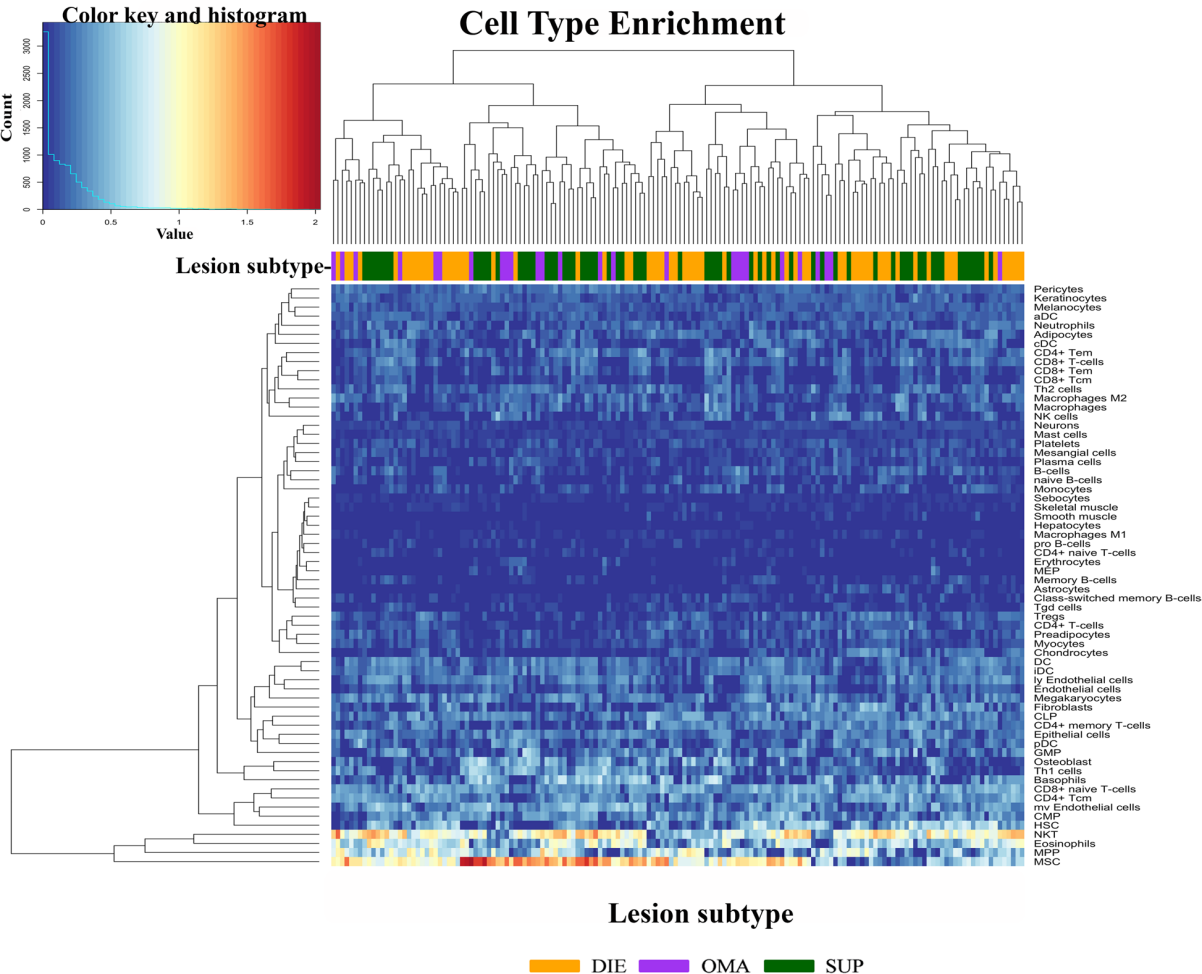
Cellular content of lesion subtypes by gene expression. Comparison of gene expression in lesions from each subtype with cell types present in the Human Cell atlas. Heatmap shows the enrichment of each cell type in each sample. All samples from all three lesion subtypes were enriched for MSC, MPP eosinophils and NK cells. Results indicate the differential gene expression profiles of the lesion subtypes are not driven by significant differences in the cell types sampled within lesions

Lastly, to estimate cell types within lesion tissue and to determine whether clustering could be driven by differences in cell type composition, gene expression profiles of each sample were compared to the Human cell atlas using xCell. A comparison of the cell type enrichment indicated the presence of a considerable stem cell component consisting of mesenchymal stem cells (MSC), multipotent progenitor cells (MPP) and hematopoietic stem cells (HSC) (Fig. 5). Samples were also enriched for immune cells, in particular NKT cells and eosinophils. The expression level of epithelial and fibroblast cells was lower but consistent across all sample types. Comparison to the human cell atlas however did not indicate that there were sufficient differences between cell type composition to drive sample clustering. The findings of this study indicate that the subtype of the lesion, rather than the menstrual stage or cell composition, may be responsible for the observed differences in gene expression profiles amongst individual lesions. These differences in gene expression profiles could potentially contribute to variations in the functional characteristics of specific lesions. However, it is important to note that the validity of identifying the cell composition of mixed tissue using will be limited by the genes that are included on the array.

### Increased ESR2 expression in OMA influences lesion response to hormones

**Fig. 6:**
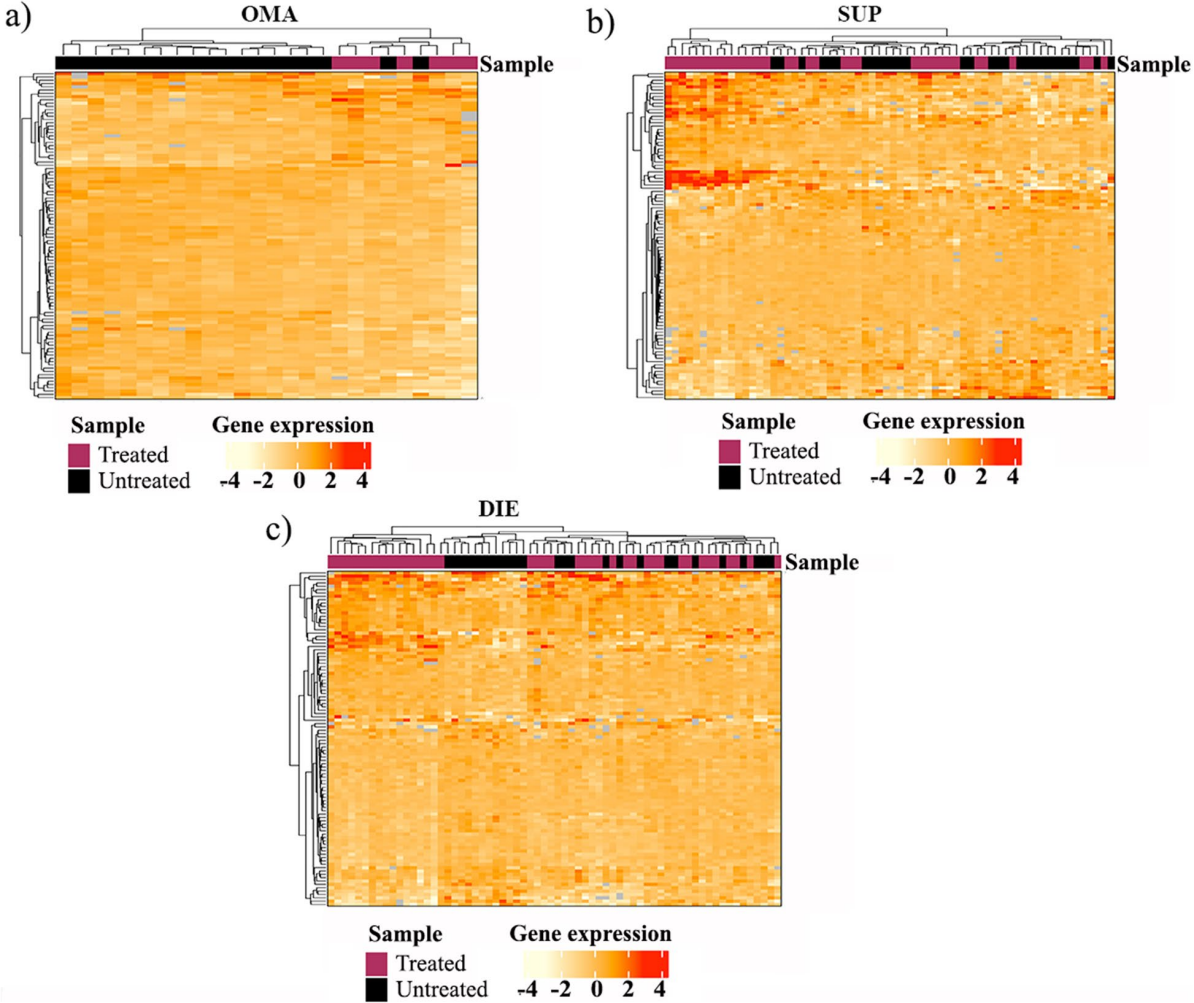
The influence of hormonal treatments on endometriosis lesion gene expression in different lesion subtypes. Heatmap clustering using the top 100 genes with variable expression was performed with samples labelled based on whether they were taking hormonal treatment prior to surgery. **a** Ovarian endometriosis revealed samples cluster based on treatment. **b** Peritoneal lesions showed a lesser degree of clustering based on treatment. **c** DIE lesions did not appear to cluster based on hormonal treatment. List of genes used for sample clustering is available in Tables S7, S8 and S9

In the absence of menstrual cycle stage effects on lesions, samples from all cycle stages were combined to test the effect of hormonal treatment (Fig. 6). Unsupervised heatmap clustering based on the top 100 genes from the DE analysis revealed that hormonally medicated OMA samples cluster separately from un-medicated OMA samples (Additional file 1: Table S8, Fig. 6a). In contrast, no clustering was observed in SUP (Additional file 1: Table S9, Fig. 6b) or DIE lesions (Additional file 1: Table S10, Fig. 6c) that could be attributed to the medication. Genome-wide differential expression analysis between medicated and non-medicated samples in each lesion subtype resulted in 569 (DIE), 836 (OMA) and 638 (SUP) nominally significant genes (*p* < 0.05).

**Fig. 7:**
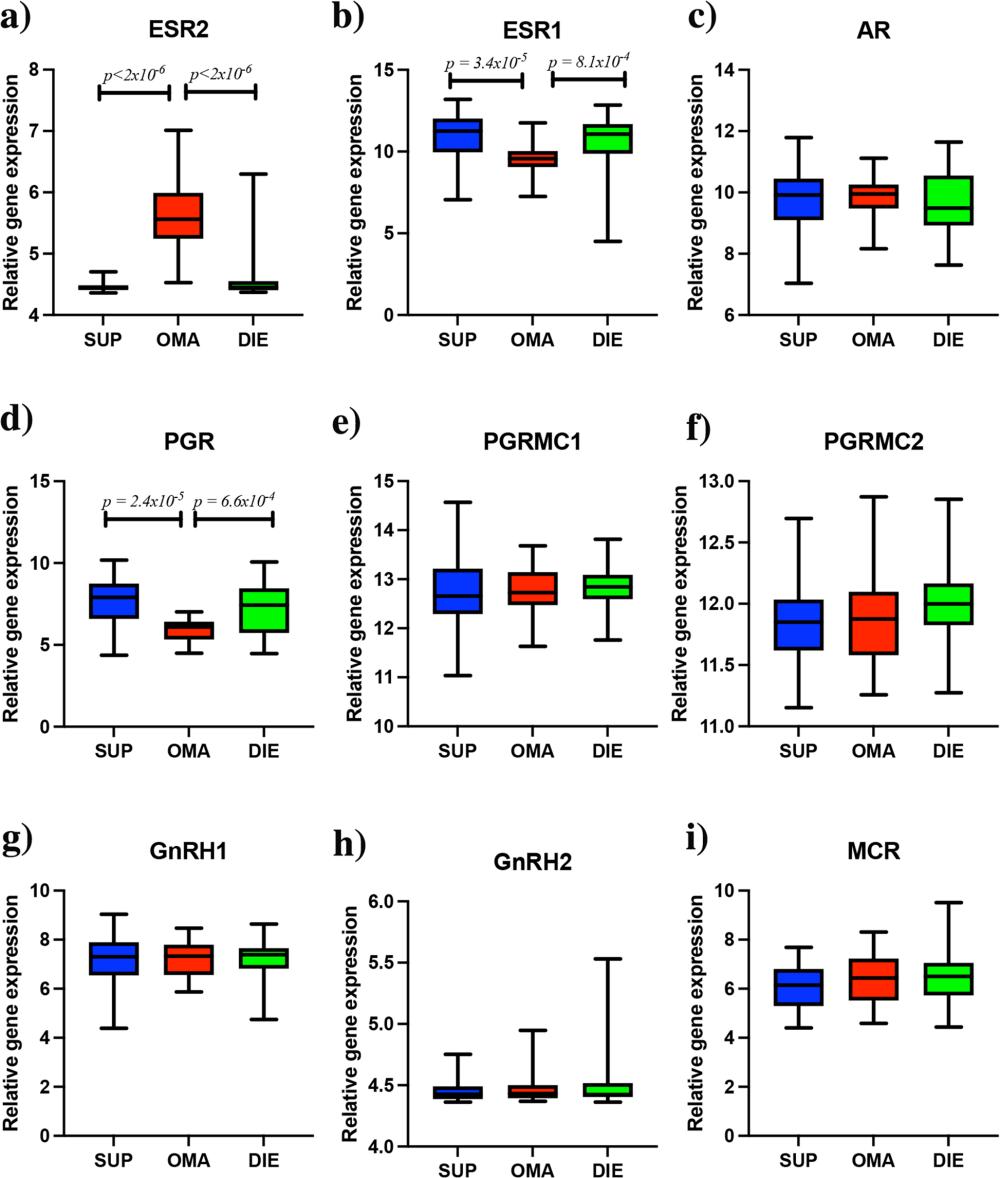
Gene expression of receptors targeted by hormonal treatments in different lesion subtypes. The level of expression was determined for hormone receptors that can be targeted by hormones used for endometriosis treatment. We identified a significantly increased expression of **a** *ESR2* in OMA compared to all other lesions and significantly decreased expression of **b** *ESR1* and **d** *PGR* was observed. No significant differences were observed for **c** *AR*, **e** *PGRMC1*, **f** *PGRMC2*, **g** *GnRH1*, **h** *GnRH2* and **i** *MCR*

The expression of target receptors for steroid hormones was evaluated by plotting their expression in both medicated and non-medicated samples and in each lesion subtype. The expression of *ESR2* (Fig. 7a), *ESR1* (Fig. 7b), *AR* (Fig. 7c), *PGR* (Fig. 7d), the progesterone membrane receptors *PGRMC1* (Fig. 7e) and *PGRMC2* (Fig. 7f), *GnRH1* (Fig. 7g), *GnRH2* (Fig. 7h) and *MCR* (Fig. 7i) was observed in each lesion subtype. A significant increase in the expression of *ESR2* (*p* < 2 × 10^−16^) was observed in the OMA lesions compared to SUP, or DIE. A significant decrease in the expression of *ESR1* (*p* = 4.42 × 10^−5^) and *PGR* (*p* = 4.85 × 10^−5^) was observed in OMA lesions compared to DIE and SUP lesions. Additional analysis of *ESR2* expression revealed that only 52 SUP and 53 DIE samples expressed *ESR2*. Together this data suggests a coordinated response to exogenous hormones in OMA and that an elevated *ESR2* expression may be important in this process. How this influence is mediated is not yet clear as changes stimulated by hormonal treatment to endogenous hormones may also impact the gene expression profiles.

### Steroid hormone co-regulation altered by hormonal medication

**Fig. 8:**
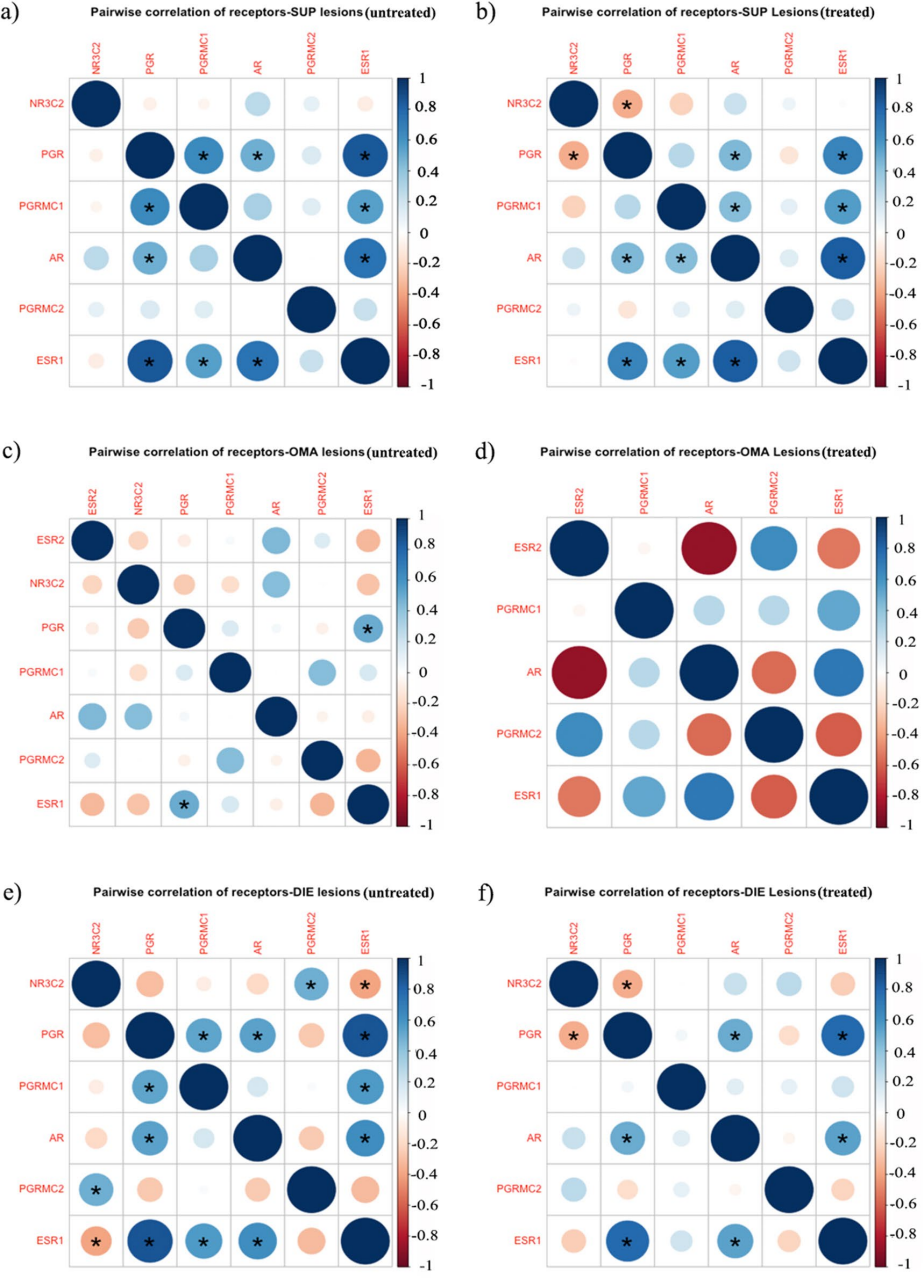
Correlation matrix displaying the correlation coefficients for the level of expression of gene pairs targeted by endometriosis treatment. Gene expression was measured in endometriotic lesion samples taken from women without treatment (untreated) and with treatment (treated). Correlations in SUP (**a**, **b**), without treatment (**a**) and with treatment (**b**) remained consistent. In contrast, the correlation between hormonal receptors in OMA (**c**, **d**) showed significant variation when comparing **c** without treatment and **d** with treatment. Similar analysis of DIE lesions (**e, f**) identified small differences in the correlation between hormone receptors both **e** without and **f** with treatment. * represents nominally significant correlations

Steroid receptors are transcription factors that modulate gene transcription by interacting with co-regulators. A pair-wise correlation between steroid receptors in SUP lesions without (Fig. 8a) and with (Fig. 8b) treatment, OMA lesions without (Fig. 8c) and with (Fig. 8d) and DIE lesions without (Fig. 8e) and with (Fig. 8f) treatment was assessed. This analysis revealed a similar correlation pattern between the SUP and DIE lesions in samples derived from patients with and without treatment, but distinct patterns in OMA samples.

In SUP lesions (Fig. 8a, b), the direction of correlation between the hormonal receptors was consistent between medicated and non-medicated samples. There was a significant positive correlation between *ESR1* and *PGR* and the membrane *PGR* receptors *PGRMC1* and *PGRMC2*. DIE lesions (Fig. 8e) revealed a significant positive correlation between *PGR* and *ESR1* as well as *PGRMC1*. *ESR1* was also correlated significantly with *AR*, but negatively with *NR3C2*. Additionally, *NR3C2* exhibited a significant positive correlation with *PGRMC2*. In hormone-medicated patients (Fig. 8f), the direction of these correlations remained consistent, with *ESR1* exhibiting a significant positive correlation with *PGR* and a negative association with *NC3R2*, and *PGR* exhibiting a positive correlation with *AR*.

In contrast, both the direction and strength of the correlation between receptors was significantly altered in OMA samples taken from medicated patients compared to non-medicated. The correlation between *ESR1* and *PGR* was positive in non-medicated samples (Fig. 8c), with no expression of *PGR* in medicated samples (Fig. 8d). A negative association between *ESR1* and *NR3C2* was observed in non-medicated (positive) and with no expression of *NR3C2* in medicated patients. There was a positive correlation between *ESR1* and *PGRMC1*. Correlations with AR were also impacted after treatment with a stronger correlation with *ESR2* (negative) and *ESR1* (positive) that were not evident in non-medicated patients. According to this study, OMA appears more sensitive to exogenous hormonal treatment than SUP or DIE, which generated distinct hormone receptor profiles after exposure.

### Co-regulated ESR2 genes is affected by hormonal medication in OMA

**Fig. 9:**
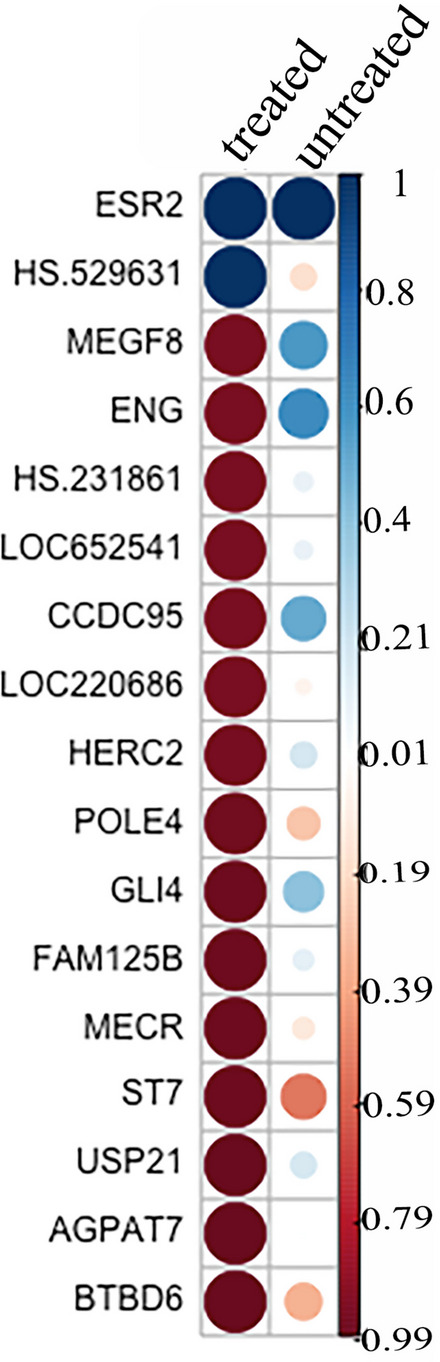
Top 20 genes correlated with *ESR2* in OMA lesions with (treated) and without treatment (untreated). The top 20 genes found to be correlated with *ESR2* expression in untreated samples were selected and their correlation after treatment was compared in OMA

To determine whether the increased expression of *ESR2* in OMA provided the potential for exogenous hormones to initiate a change in gene expression profiles, the top 20 genes correlated with *ESR2* expression were evaluated (Fig. 9), and the direction and strength was compared between medicated and non-medicated patients. This analysis revealed a significant alteration in the genes correlated with *ESR2* in OMA samples from medicated and non-medicated patients. At least 10 of these 20 genes, including *MEGF8*, *ENG, CCDC95*, *HERC2*, and *GLI4*, exhibited an opposite direction of action in medicated and non-medicated samples (Fig. 9), indicating that genes associated with *ESR2* are significantly altered by medication.

## Discussion

Endometriosis is a heterogeneous disease with inadequate treatment options. Surgical procedures to remove lesions are challenging and can result in adverse effects. Medical treatment involves the systemic suppression of oestrogen, a process accompanied by significant side effects and variable effectiveness in reducing endometriosis-related symptoms. Neither are adequate. In this study, gene expression profiles in endometriotic lesions and the influence of hormone treatment were evaluated. This study demonstrated that subtypes of endometriotic lesions have distinct expression profiles and that exogenous hormones directly affect OMA, but not SUP or DIE. The overabundance of *ESR2* in OMA and the altered gene expression profiles in hormonally medicated samples suggest OMA lesions are directly amenable to hormonal treatments.

Gene expression can be influenced by technical and biological factors. Ancestry has been shown to result in significant differences in gene expression [25] and gene expression in the endometrium is dynamic throughout the menstrual cycle [26]. In this study, gene expression levels were compared to a previously published endometrium microarray dataset in order to serve as a benchmark for the database containing lesions and hormonally medicated data. This analysis confirmed that the expression of genes in the two datasets was highly correlated, despite the fact that tissue was obtained from patients of different ancestry. The degree of correlation was further increased once genes significantly regulated across the menstrual cycle were taken into account providing confidence in the quality and consistency of the dataset.

In hormone-treated women, obtaining adequate endometrial samples for RNA isolation can be difficult. Hormones result in an inert endometrium with minimal accessible tissue for harvest. Consequently, their influence on the endometrium is not well understood. Our genome-wide analysis revealed that hormones significantly influenced the endometrium and that these differences manifest as significantly distinct functions. Hormone-medicated samples exhibited significantly fewer differentially expressed genes compared to the secretory stage than the proliferative stage. The proliferative stage is exposed to endogenous oestrogen, while the secretory stage is exposed to both oestrogen and progesterone. In this analysis, 14 out of 21 (67%) of the hormonal treatments used in this analysis were combined oestrogen and progesterone-based treatments, similar to the exposure that might occur during the secretory stage.

In contrast to the endometrium, gene expression profiles in endometriotic lesions were not significantly affected by the menstrual stage. This was regardless of whether lesions were considered collectively or as distinct subtypes. It was previously believed that endometriosis lesions possess hormone receptors that allow them to respond to circulating steroid hormones and this may contribute to the cyclical pain experienced by women with the disease [12, 13, 27]. A proteomic study failed to distinguish lesions on the basis of the menstrual stage [28]. Previous research has shown that hormone receptor expression may vary greatly amongst lesions [29] and that inflammation can affect hormone receptor expression [14]. It is thus possible that the response of individual lesions to hormones may differ based on individual characteristics influenced by their age or exposure to inflammatory conditions.

In contrast to the menstrual cycle, gene expression profiles could be distinguished based on lesion subtypes and that OMA lesions displayed a significantly distinct profile compared to both DIE and SUP lesions. Similar clustering based on lesion subtype was also observed in previous work with this dataset [30]. Lesion clustering could be driven by cell types present within the sample. Surgically removed endometriosis lesions will contain surrounding tissue that may influence gene expression profiles. To determine whether different cell types were overrepresented, a cell enrichment analysis revealed no clustering based on cell type. Interestingly, an abundance of stem cell-like cells and immune cells were identified, contributing to the complex microenvironment of these lesions. As anticipated, epithelial and fibroblast cells were also enriched in the tissue, but not within a specific subtype.

Additionally, OMA gene expression profiles were significantly altered, with medicated samples clustering apart from non-medicated samples. Hormone levels were observed to be higher in ovarian lesions [31]. In contrast, no such separation was observed in the SUP or DIE. Hormonal treatments for endometriosis primarily target *ESR1*, *ESR2*, or *PGR* and have the highest affinity for these receptors; however, due to the structural similarity of hormone receptors, off-target effects at other receptors are possible [32]. It was observed of these receptors that *ESR2* showed the greatest variation between OMA and other lesions, creating the potential for hormones to directly influence the microenvironment of OMA through increased *ESR2* expression.

In support of OMA-specific response to hormones both hormone receptors and co-regulated gene expression with *ESR2* varied significantly in medicated samples compared to un-medicated samples in OMA, but not SUP or DIE. *ESR2* is a nuclear transcription factor responsible for the regulation of numerous downstream genes. After hormonal treatment, OMA lesions exhibited a significantly different set of genes correlated with the *ESR2* suggesting a coordinated response that does not occur in other lesion types. Previous research has demonstrated the downregulation of genes in response to treatment [33]. This suggests that hormone treatments can directly influence OMA by altering gene expression and, subsequently, cellular behaviour. Further investigation is warranted to establish a causal relationship.

Despite the presence of both *ESR1* and *ESR2* in the endometrium [34], *ESR2* expression predominates in the ovary [35, 36]. An abundance of *ESR1* over *ESR2* in OMA has been reported [37], although the relative ratio of ESR1 to ESR2 has been observed to be lower in OMA and OMA-derived stromal cells [38, 39]. It is believed that increased *ESR2* expression downregulates *ESR1* by binding to alternative promoter regions [40]. Previous research has demonstrated a substantial increase in OMA-induced stromal cell expression of *ESR2* in comparison to healthy eutopic stromal cells [41]. It has been hypothesised that differential methylation is a major mechanism driving *ESR2* upregulation in stromal cells [41], although there appears to be little research performed in epithelial cells. Increased *ESR2* has been linked to increased proliferation, apoptosis, inflammation, and pain transmission in endometriosis [42, 43]. *ESR2* interacts with cytosolic inflammasome components to increase interleukin (IL)-1B in mouse models [44], and ESR2-immunoreactive macrophages contribute to inflammation and mediate nerve growth [45].

It has been proposed that agents that inhibit ESR2-mediated inflammation could represent promising new treatment options [34]. A recently developed *ESR2* ligand, chloroindazole, can prevent lesion formation in mice [46], as can the SRC-1 inhibitor bufalin, which induces *ESR2* protein degradation and endometriotic epithelial cell apoptosis [47]. It may be possible to target these cells specifically to OMA lesions, or to offer mixed treatment options that activate and target *ESR2* in OMAs.

Although this study identifies an OMA-specific response to hormonal treatments by oestrogen-suppressive agents, it has limitations. Firstly, larger sample sizes would allow the possibility to identify subtle differences in gene expression. Although it is not as strongly powered as hoped, it still represents sample sizes greater than many published studies and does allow the identification of significant differences that reveal important insights and are largely consistent and build upon previous data. Due to the small sample sizes, it was not possible to distinguish effects by distinct hormonal medications. By analysing each hormone treatment separately in a higher-powered study, it may be possible to gain a better understanding of how hormones influence endometriosis lesions.

## Conclusions

In summary, we identified significantly different gene expression profiles for lesions of different subtypes. OMA lesions appear to be significantly different from the other two subtypes, harbour altered steroid receptor expression and responded to hormonal medication differently. It is possible therefore that OMA tissue may be more sensitive to hormone treatment; hence, women with OMA lesions may get more benefit from customized hormonal therapies. Further studies are required to assess causality and determine the mechanism by which this variance is produced.

### Supplementary Information

**Additional file 1:** **Table S1.** Hormonal treatment taken by the women within 3 months prior to surgery. **Table S2.** Top 30 genes (from total of 142 following Bonferroni correction *p*<3.54x10-06) that were significantly different between proliferative and secretory phases of samples obtained from patient endometrium. **Table S3.** Genes that were significantly differentially expressed (following Bonferroni correction *p*<3.41x10^-06^) between proliferative and medicated samples obtained from patient endometrium. **Table S4.** Genes that were significantly different (following FDR correction) between secretory and medicated samples obtained from patient endometrium. **Table S5.** Top 50 genes that were significantly differently expressed (following FDR correction) between DIE and SUP samples. **Table S6.** Top 50 genes that were significantly differently expressed (following Bonferroni and FDR correction) between OMA and SUP samples. **Table S7.** Top 50 genes that were significantly differently expressed (following Bonferroni and FDR correction) between OMA and DIE samples. **Table S8.** Top 100 genes that were significantly (nominal *p*<0.05) different between medicated and non-medicated samples in OMA. **Table S9.** Top 100 genes that were significantly (nominal *p*<0.05)) different between medicated and non-medicated samples in SUP. **Table S10.** Top 100 genes that were significantly (nominal *p*<0.05)) different between medicated and non-medicated samples in DIE.

## Data Availability

The data underlying this article is available from the Gene Expression Omnibus (GEO) GSE141549 [19, 20].
