# Supplementary material for: Gene expression profiles separate endometriosis lesion subtypes and indicate a sensitivity of endometrioma to estrogen suppressive treatments through elevated ESR2 expression
Source: BMC Med. 2023 Nov 23;21:460. doi: 10.1186/s12916-023-03166-1 (PMC10666321; doi:10.1186/s12916-023-03166-1)
Supplement: Supplementary file 1 — Additional file 1: Table S1. Hormonal treatment taken by the women within 3 months prior to surgery. Table S2. Top 30 genes (from total of 142 following Bonferroni correction p<3.54x10^-06^) that were significantly different between proliferative and secretory phases of samples obtained from patient endometrium. Table S3. Genes that were significantly differentially expressed (following Bonferroni correction p<3.41x10^-06^) between proliferative and medicated samples obtained from patient endometrium. Table S4. Genes that were significantly different (following FDR correction) between secretory and medicated samples obtained from patient endometrium. Table S5. Top 50 genes that were significantly differently expressed (following FDR correction) between DIE and SUP samples. Table S6. Top 50 genes that were significantly differently expressed (following Bonferroni and FDR correction) between OMA and SUP samples. Table S7. Top 50 genes that were significantly differently expressed (following Bonferroni and FDR correction) between OMA and DIE samples. Table S8. Top 100 genes that were significantly (nominal p<0.05) different between medicated and non-medicated samples in OMA. Table S9. Top 100 genes that were significantly (nominal p<0.05)) different between medicated and non-medicated samples in SUP. Table S10. Top 100 genes that were significantly (nominal p<0.05)) different between medicated and non-medicated samples in DIE. [file 12916_2023_3166_MOESM1_ESM.docx]

**Supplementary Tables**

**Table S1: Hormonal treatment taken by the women within 3 months prior to surgery.**

| **Hormonal treatment** | **Endometrium + Endo** | **Endometrium - Endo** | **SUP** | **OMA** | **DIE** |
| --- | --- | --- | --- | --- | --- |
| Diane Nova | 0 | 0 | 1 | 0 | 3 |
| e-pilleri | 0 | 0 | 0 | 0 | 3 |
| EVRA | 0 | 0 | 0 | 0 | 3 |
| Femoden | 1 | 0 | 2 | 0 | 3 |
| Harmonet | 0 | 2 | 0 | 0 | 1 |
| Meliane | 1 | 1 | 0 | 0 | 0 |
| Mercilon | 1 | 0 | 3 | 1 | 2 |
| Microgynon | 1 | 0 | 1 | 0 | 1 |
| Minulet | 0 | 0 | 1 | 0 | 1 |
| Mirelle | 1 | 0 | 4 | 0 | 0 |
| Mirena | 1 | 2 | 2 | 0 | 3 |
| Nuvaring | 1 | 0 | 3 | 0 | 1 |
| Primolut | 1 | 0 | 0 | 0 | 3 |
| Primulot Nor | 1 | 0 | 0 | 0 | 1 |
| Procren depot | 2 | 0 | 0 | 1 | 6 |
| Provera | 1 | 0 | 1 | 0 | 2 |
| Yasmin | 7 | 0 | 16 | 4 | 7 |
| Yasminelle | 1 | 0 | 0 | 0 | 0 |
| Zoladex caps | 1 | 0 | 1 | 1 | 0 |
| Endometrium+Endo=Endometrial samples from women with endometriosis  Endometrium-Endo= Endometrial samples from women without endometriosis  SUP= Superficial peritoneal lesions; OMA=Ovarian endometrioma; DIE= Deep infiltrated endometriosis | | | | | |

Table S2: Top 30 genes (from total of 142 following Bonferroni correction p<3.54x10-06) that were significantly different between proliferative and secretory phases of samples obtained from patient endometrium.

| **Genes** | **logFC** | **AveExpr** | **P.Value** | **FDR** |
| --- | --- | --- | --- | --- |
| GJA4 | 2.61 | -0.34 | 1.38E-09 | 1.18E-05 |
| GPBAR1 | -2.72 | 0.001 | 2.19E-09 | 1.18E-05 |
| PLEKHB1 | 2.09 | 0.153 | 2.37E-09 | 1.18E-05 |
| SDK2 | 2.69 | 0.63 | 5.51E-09 | 1.88E-05 |
| ST7OT1 | 1.05 | -0.08 | 6.31E-09 | 1.88E-05 |
| PODXL2 | 1.97 | 0.69 | 7.62E-09 | 1.89E-05 |
| ATP8B3 | 2.98 | 1.02 | 1.27E-08 | 2.70E-05 |
| RAPH1 | -1.14 | -0.28 | 1.45E-08 | 2.71E-05 |
| AKAP13 | -1.34 | -0.11 | 2.42E-08 | 4.01E-05 |
| DERA | -0.74 | 0.51 | 3.21E-08 | 4.79E-05 |
| DKK1 | -3.94 | 0.02 | 3.84E-08 | 4.88E-05 |
| CD44 | -0.96 | -0.09 | 4.01E-08 | 4.88E-05 |
| NPAS3 | 2.92 | 0.95 | 4.27E-08 | 4.88E-05 |
| OGDHL | 2.24 | 0.62 | 5.25E-08 | 4.88E-05 |
| LOC645558 | 1.07 | 0.46 | 5.30E-08 | 4.88E-05 |
| HS.336675 | 1.75 | 0.45 | 5.49E-08 | 4.88E-05 |
| APOD | -2.03 | -0.66 | 5.73E-08 | 4.88E-05 |
| ALDH1A3 | -3.09 | -0.62 | 6.08E-08 | 4.88E-05 |
| GPER | 2.77 | -0.63 | 6.21E-08 | 4.88E-05 |
| MGST1 | -1.55 | 0.56 | 6.65E-08 | 4.96E-05 |
| PCSK5 | 1.46 | 0.02 | 8.03E-08 | 5.70E-05 |
| C13ORF33 | -3.16 | -1.12 | 8.73E-08 | 5.92E-05 |
| MYB | 2.16 | 0.96 | 9.24E-08 | 5.99E-05 |
| LOC152485 | 0.92 | -0.30 | 1.05E-07 | 6.52E-05 |
| IRS2 | -1.91 | -0.44 | 1.22E-07 | 7.09E-05 |
| MSX2 | 2.32 | 1.447 | 1.24E-07 | 7.09E-05 |
| SLC47A1 | 3.33 | 0.392 | 1.41E-07 | 7.78E-05 |
| MYL6B | 0.85 | 0.264 | 1.53E-07 | 8.04E-05 |
| VANGL2 | 1.46 | 0.032 | 1.59E-07 | 8.04E-05 |
| BCL11A | 1.64 | 0.222 | 1.62E-07 | 8.04E-05 |
| logFC = log fold change  AveExpr = average gene expression | | | | |

Table S3: Genes that were significantly differentially expressed (following Bonferroni correction p<3.41x10^-06^) between proliferative and medicated samples obtained from patient endometrium.

| **Genes** | **logFC** | **AveExpr** | **P.Value** | **FDR** |
| --- | --- | --- | --- | --- |
| RARB | 1.379 | -0.426 | 1.54E-07 | 0.0022 |
| FAM86B1 | -0.870 | 0.864 | 9.70E-07 | 0.0050 |
| OTUB2 | -1.22 | 0.989 | 1.12E-06 | 0.0050 |
| FAM107A | 2.301 | -1.102 | 1.37E-06 | 0.0050 |
| STOM | 0.920 | -0.827 | 1.85E-06 | 0.0054 |
| C19ORF48 | -0.934 | 0.8306 | 3.33E-06 | 0.0058 |
| logFC = log fold change  AveExpr = average gene expression | | | | |

Table S4: Genes that were significantly different (following FDR correction) between secretory and medicated samples obtained from patient endometrium.

| **Genes** | **logFC** | **AveExpr** | **P.Value** | **FDR** |
| --- | --- | --- | --- | --- |
| ITM2C | 0.829 | -0.297 | 4.64E-06 | 0.029 |
| RARB | 1.285 | -0.472 | 4.78E-06 | 0.029 |
| MGST1 | -1.280 | 0.600 | 5.99E-06 | 0.029 |
| LTBP3 | 0.638 | -0.466 | 1.70E-05 | 0.032 |
| CAMK1D | 0.918 | -0.729 | 1.99E-05 | 0.032 |
| EFNB1 | 1.104 | -0.569 | 2.08E-05 | 0.032 |
| L3MBTL3 | 0.654 | -0.236 | 2.41E-05 | 0.032 |
| DLG4 | 0.789 | -0.424 | 2.51E-05 | 0.032 |
| FAM125B | 0.612 | -0.426 | 2.58E-05 | 0.032 |
| SLC22A17 | 0.761 | -0.667 | 2.70E-05 | 0.032 |
| SPTLC3 | -1.452 | 1.063 | 2.75E-05 | 0.032 |
| GOLPH4 | 0.849 | -0.540 | 2.76E-05 | 0.032 |
| XTP3TPA | -0.669 | 0.556 | 2.99E-05 | 0.032 |
| CAMK2N1 | 0.8680 | -0.304 | 3.05E-05 | 0.032 |
| RSC1A1 | -0.913 | 0.451 | 3.76E-05 | 0.036 |
| MMAA | -0.692 | 0.453 | 4.44E-05 | 0.040 |
| MAPKAP1 | -0.644 | 0.303 | 4.90E-05 | 0.040 |
| KRBA1 | 0.5531 | -0.17 | 5.05E-05 | 0.040 |
| DKFZP564J0863 | -0.712 | 0.452 | 5.50E-05 | 0.040 |
| FLJ21986 | 1.009 | -0.654 | 5.54E-05 | 0.040 |
| HS.145049 | -0.554 | 0.348 | 5.95E-05 | 0.041 |
| KRT18 | -1.018 | 0.451 | 6.52E-05 | 0.042 |
| TMEM50A | -0.439 | 0.234 | 7.07E-05 | 0.042 |
| ACOT4 | -0.960 | 0.893 | 7.41E-05 | 0.042 |
| FLNC | 1.237 | -1.391 | 7.54E-05 | 0.042 |
| C3ORF59 | 1.037 | -0.956 | 7.75E-05 | 0.042 |
| BCL11A | 1.132 | 0.072 | 7.82E-05 | 0.042 |
| PMM2 | -0.498 | 0.466 | 8.42E-05 | 0.043 |
| GINS3 | -0.925 | 0.723 | 8.54E-05 | 0.043 |
| RANGRF | 0.429 | 0.159 | 0.00010236 | 0.048 |
| CD74 | 0.798 | -0.597 | 0.0001031 | 0.048 |
| SYNGR1 | 0.757 | -0.408 | 0.00010649 | 0.048 |
| FCGBP | 1.278 | 0.664 | 0.00011398 | 0.048 |
| NAB2 | 0.549 | -0.105 | 0.00011545 | 0.048 |
| PHF2 | 0.334 | -0.151 | 0.00011838 | 0.048 |
| TMEM47 | 0.953 | -1.072 | 0.0001234 | 0.048 |
| DNAJC18 | 0.598 | -0.600 | 0.0001309 | 0.048 |
| KLHL5 | 0.600 | -0.552 | 0.00013246 | 0.048 |
| HS.99472 | 1.341 | -1.699 | 0.00013679 | 0.048 |
| HS.60257 | -1.238 | 1.019 | 0.0001499 | 0.048 |
| THRA | 0.918 | -0.417 | 0.00015239 | 0.048 |
| CROCC | 0.684 | -0.312 | 0.0001558 | 0.048 |
| LOC152485 | 0.659 | -0.372 | 0.00015903 | 0.048 |
| SYT11 | 0.905 | -0.435 | 0.00016127 | 0.048 |
| SH3GLB1 | -0.46 | 0.114 | 0.00016863 | 0.048 |
| PDE8B | 1.280 | -0.576 | 0.00017157 | 0.048 |
| PBX2 | 0.403 | -0.180 | 0.00017324 | 0.048 |
| CENTD3 | 0.543 | -0.340 | 0.00017942 | 0.048 |
| JAM3 | 0.608 | -0.647 | 0.00018374 | 0.048 |
| CLIC6 | 1.538 | -0.373 | 0.0001848 | 0.048 |
| BTBD6 | -0.475 | 0.162 | 0.00018996 | 0.048 |
| MGST1 | -1.142 | 0.602 | 0.00019544 | 0.048 |
| EXOC4 | 0.208 | -0.103 | 0.00019935 | 0.048 |
| HS.336675 | 0.877 | 0.120 | 0.00020041 | 0.048 |
| STARD10 | 0.536 | 0.001 | 0.00020299 | 0.048 |
| APEG1 | 1.356 | -1.218 | 0.00020536 | 0.048 |
| CCDC64 | -1.132 | 0.366 | 0.00020707 | 0.048 |
| ASPN | 1.969 | -2.166 | 0.00020782 | 0.048 |
| LOC728069 | 0.909 | -0.297 | 0.00021089 | 0.048 |
| B3GNT1 | 0.5629 | -0.445 | 0.00021203 | 0.048 |
| ETNK2 | 1.045 | -0.421 | 0.0002125 | 0.048 |
| VNN1 | -1.924 | 1.544 | 0.00022193 | 0.048 |
| MTAC2D1 | -1.198 | 0.778 | 0.00022293 | 0.048 |
| MTHFS | -0.546 | 0.428 | 0.00022537 | 0.048 |
| COMMD6 | 0.302 | -0.208 | 0.00022576 | 0.048 |
| MGST1 | -1.461 | 0.678 | 0.00022882 | 0.048 |
| SAT2 | 0.303 | -0.163 | 0.00023034 | 0.048 |
| LOC644330 | -0.306 | 0.184 | 0.0002321 | 0.048 |
| SERPINB6 | 0.231 | -0.019 | 0.00023496 | 0.048 |
| DDT | -0.367 | 0.278 | 0.00023824 | 0.048 |
| ANXA2P3 | -0.926 | 0.469 | 0.00024327 | 0.048 |
| PCGF2 | 0.421 | -0.119 | 0.00024568 | 0.048 |
| WNT7A | 1.506 | 0.425 | 0.00024607 | 0.048 |
| HERC5 | 1.020 | -0.870 | 0.00024648 | 0.048 |
| ANXA2P1 | -0.941 | 0.421 | 0.00024782 | 0.048 |
| ZCCHC12 | 2.096 | -0.723 | 0.00025103 | 0.048 |
| CACNA1H | 1.115 | -0.513 | 0.00025147 | 0.048 |
| logFC = log fold change  AveExpr = average gene expression | | | | |

Table S5: Top 50 genes that were significantly differently expressed (following FDR correction) between DIE and SUP samples.

| **Genes** | **logFC** | **AveExpr** | **P.Value** | **FDR** |
| --- | --- | --- | --- | --- |
| TUT1 | -0.63973 | -0.10081 | 1.49E-07 | 0.0012 |
| HOXC8 | -2.05789 | 0.26079 | 1.73E-07 | 0.0012 |
| TMEM136 | -0.56175 | 0.02188 | 2.65E-07 | 0.0012 |
| C19ORF25 | -0.57277 | -0.07625 | 5.03E-07 | 0.0016 |
| DBP | -1.41004 | -0.28012 | 9.52E-07 | 0.0016 |
| DNAJA4 | 0.80066 | 0.02230 | 1.10E-06 | 0.0016 |
| WFDC1 | 1.93178 | 0.37371 | 1.21E-06 | 0.0016 |
| ANKRD57 | 0.57266 | 0.16175 | 1.23E-06 | 0.0016 |
| PDE6B | -0.98947 | 0.00022 | 1.25E-06 | 0.0016 |
| HOXC6 | -2.16348 | 0.58467 | 1.27E-06 | 0.0016 |
| COQ10B | 0.52716 | 0.06947 | 1.36E-06 | 0.0016 |
| TK2 | -0.49463 | 0.01251 | 1.67E-06 | 0.0016 |
| ASXL1 | -0.36804 | 0.02528 | 1.68E-06 | 0.0016 |
| UBE2L6 | -0.37046 | -0.00145 | 1.85E-06 | 0.0016 |
| LOC647000 | -0.47728 | -0.02359 | 1.95E-06 | 0.0016 |
| ZNF467 | -0.69701 | -0.14017 | 2.22E-06 | 0.0016 |
| BTN3A3 | -0.70290 | 0.02260 | 2.25E-06 | 0.0016 |
| TAF5L | 0.80453 | 0.00903 | 2.27E-06 | 0.0016 |
| SRCRB4D | -1.43349 | -0.02605 | 2.31E-06 | 0.0016 |
| CALD1 | 1.88238 | 0.64386 | 2.35E-06 | 0.0016 |
| CDC37L1 | 0.53425 | 0.08986 | 2.38E-06 | 0.0016 |
| TMEM42 | -0.59146 | -0.0321 | 2.73E-06 | 0.0017 |
| EFNB3 | -1.44616 | -0.09187 | 3.01E-06 | 0.0017 |
| ADAR | -0.23409 | -0.02251 | 3.05E-06 | 0.0017 |
| ISCA2 | -0.48909 | -0.02701 | 3.19E-06 | 0.0017 |
| RTN4R | -1.19317 | -0.18069 | 3.25E-06 | 0.0017 |
| ZNF792 | -0.90292 | -0.09085 | 3.50E-06 | 0.0017 |
| CUL7 | -0.65938 | -0.12456 | 3.62E-06 | 0.0017 |
| LRRC29 | -0.53411 | -0.04064 | 3.65E-06 | 0.0017 |
| CYBASC3 | -0.64267 | 0.07061 | 3.76E-06 | 0.0017 |
| CCDC8 | -1.04688 | 0.18189 | 3.79E-06 | 0.0017 |
| ZNF142 | -0.30611 | -0.04692 | 4.08E-06 | 0.0018 |
| CACNB2 | 1.34353 | 0.17757 | 4.54E-06 | 0.0019 |
| FAM26B | -0.85361 | 0.11554 | 5.02E-06 | 0.0020 |
| PURB | 0.50381 | -0.07092 | 5.12E-06 | 0.0020 |
| VAV1 | -1.08784 | 0.08658 | 6.03E-06 | 0.0022 |
| SUSD3 | -1.45942 | 0.38308 | 6.04E-06 | 0.0022 |
| SH2D3C | -0.58775 | -0.05525 | 6.27E-06 | 0.0022 |
| IMP3 | -0.32188 | -0.08230 | 6.35E-06 | 0.0022 |
| GJA7 | 0.58352 | 0.05307 | 6.59E-06 | 0.0022 |
| CAMK2N1 | 0.93079 | 0.17930 | 6.60E-06 | 0.0022 |
| CORO2B | -0.85787 | -0.10271 | 6.65E-06 | 0.0022 |
| C19ORF39 | -0.90337 | -0.02477 | 6.96E-06 | 0.0022 |
| CYCS | 0.63762 | 0.02354 | 7.28E-06 | 0.0023 |
| CAD | -0.47925 | -0.03172 | 7.55E-06 | 0.0023 |
| PARS2 | -0.64520 | -0.17161 | 8.63E-06 | 0.0026 |
| ZKSCAN4 | -0.85551 | -0.01365 | 8.96E-06 | 0.0026 |
| DMN | 2.29304 | 0.59052 | 9.00E-06 | 0.0026 |
| POPDC2 | 1.65384 | 0.44310 | 9.12E-06 | 0.0026 |
| C18ORF17 | -0.60715 | -0.04953 | 9.26E-06 | 0.0026 |
| logFC = log fold change  AveExpr = average gene expression | | | | |

Table S6: Top 50 genes that were significantly differently expressed (following Bonferroni and FDR correction) between OMA and SUP samples.

| **Genes** | **logFC** | **AveExpr** | **P.Value** | **FDR** |
| --- | --- | --- | --- | --- |
| NR5A1 | -3.1289 | 0.5291 | 1.41E-19 | 2.00E-15 |
| AQP11 | -3.3348 | 0.5117 | 8.11E-16 | 5.74E-12 |
| SERPINE2 | -2.0866 | 0.6015 | 5.70E-15 | 2.69E-11 |
| FAM98A | -0.7654 | 0.0841 | 3.52E-13 | 1.25E-09 |
| PPAP2A | 1.2390 | -0.2991 | 7.54E-13 | 2.13E-09 |
| NEFH | -2.9778 | 0.4076 | 1.77E-12 | 4.19E-09 |
| PLVAP | 2.2576 | -0.0149 | 3.19E-12 | 6.04E-09 |
| FLJ37440 | 2.7086 | -0.4281 | 3.41E-12 | 6.04E-09 |
| BAIAP2 | 1.4848 | -0.2359 | 4.91E-12 | 7.73E-09 |
| COLEC11 | -2.8719 | 0.3258 | 8.61E-12 | 1.22E-08 |
| ST3GAL4 | -1.6745 | 0.3922 | 1.15E-11 | 1.43E-08 |
| CYP11A1 | -2.8881 | 0.2422 | 1.21E-11 | 1.43E-08 |
| PODXL | 1.8588 | -0.1978 | 1.39E-11 | 1.51E-08 |
| TRIM24 | -1.0325 | 0.1356 | 2.02E-11 | 2.05E-08 |
| FHL2 | -1.5979 | 0.4689 | 2.46E-11 | 2.32E-08 |
| TSHZ1 | -1.2073 | 0.1413 | 2.95E-11 | 2.61E-08 |
| LEPREL1 | -1.2511 | 0.3103 | 3.48E-11 | 2.85E-08 |
| UBXD6 | -2.1051 | 0.1472 | 3.70E-11 | 2.85E-08 |
| PGM1 | -0.8298 | 0.1214 | 3.82E-11 | 2.85E-08 |
| PPAP2A | 1.4918 | -0.2827 | 4.34E-11 | 3.07E-08 |
| ING2 | -1.5444 | 0.0654 | 8.76E-11 | 5.90E-08 |
| RWDD4A | -0.9161 | 0.1138 | 9.25E-11 | 5.96E-08 |
| ITPK1 | 0.8039 | -0.1325 | 1.09E-10 | 6.36E-08 |
| ITGB4 | 1.6135 | -0.2237 | 1.10E-10 | 6.36E-08 |
| CKAP5 | -0.6200 | 0.0359 | 1.12E-10 | 6.36E-08 |
| ALDH1A2 | 2.0347 | -0.3284 | 1.47E-10 | 8.02E-08 |
| RSPO3 | 3.2538 | -0.0431 | 1.87E-10 | 9.79E-08 |
| HOXA5 | 2.6717 | -0.4279 | 1.96E-10 | 9.90E-08 |
| HSD11B1 | -2.9457 | 0.1390 | 2.29E-10 | 1.12E-07 |
| PPAP2A | 1.3710 | -0.3588 | 2.50E-10 | 1.18E-07 |
| CRIP1 | 1.2774 | -0.3689 | 3.60E-10 | 1.65E-07 |
| MAG | -2.4950 | 0.6114 | 3.72E-10 | 1.65E-07 |
| HOXB2 | 2.0931 | -0.4547 | 4.91E-10 | 2.06E-07 |
| SIPA1L2 | -1.0841 | 0.2340 | 4.95E-10 | 2.06E-07 |
| HS.306410 | 2.4486 | -0.4350 | 7.17E-10 | 2.90E-07 |
| CD9 | 0.9803 | -0.0768 | 7.39E-10 | 2.91E-07 |
| IQCG | -2.0521 | 0.2261 | 1.01E-09 | 3.83E-07 |
| SCARA3 | 1.7387 | 0.0655 | 1.03E-09 | 3.83E-07 |
| KCTD14 | 2.2705 | -0.5765 | 1.15E-09 | 4.17E-07 |
| CTNNAL1 | -1.2606 | 0.0751 | 1.40E-09 | 4.86E-07 |
| FBLN2 | 1.4175 | -0.2745 | 1.41E-09 | 4.86E-07 |
| SCARA3 | 2.5045 | 0.0349 | 1.47E-09 | 4.94E-07 |
| SEMA5A | 1.8300 | -0.3583 | 1.99E-09 | 6.56E-07 |
| EBF3 | 2.8093 | -0.5226 | 2.54E-09 | 8.19E-07 |
| CAMK1G | -1.9385 | 0.5714 | 3.08E-09 | 9.69E-07 |
| C1ORF54 | 1.1705 | 0.0059 | 3.19E-09 | 9.76E-07 |
| PDE4A | 1.0459 | -0.1064 | 3.24E-09 | 9.76E-07 |
| WNK3 | -1.3217 | 0.2238 | 4.31E-09 | 1.27E-06 |
| MSH6 | -0.6416 | 0.0977 | 5.52E-09 | 1.59E-06 |
| SCARA3 | 1.9799 | 0.1057 | 5.80E-09 | 1.64E-06 |
| logFC = log fold change  AveExpr = average gene expression | | | | |

Table S7: Top 50 genes that were significantly differently expressed (following Bonferroni and FDR correction) between OMA and DIE samples.

| **Genes** | **logFC** | **AveExpr** | **P.Value** | **FDR** |
| --- | --- | --- | --- | --- |
| NR5A1 | 3.3695 | 0.5291 | 1.41E-19 | 2.00E-15 |
| AQP11 | 3.7468 | 0.5117 | 8.11E-16 | 5.74E-12 |
| CYP11A1 | 3.7602 | 0.2422 | 5.70E-15 | 2.69E-11 |
| UBXD6 | 2.6979 | 0.1472 | 3.52E-13 | 1.25E-09 |
| COLEC11 | 3.4375 | 0.3258 | 7.54E-13 | 2.13E-09 |
| PPAP2A | -1.3517 | -0.2991 | 1.77E-12 | 4.19E-09 |
| MAG | 3.1262 | 0.6114 | 3.19E-12 | 6.04E-09 |
| RPP25 | 2.0710 | 0.2581 | 3.41E-12 | 6.04E-09 |
| CKAP5 | 0.7295 | 0.0359 | 4.91E-12 | 7.73E-09 |
| SERPINE2 | 1.8771 | 0.6015 | 8.61E-12 | 1.22E-08 |
| NEFH | 3.1567 | 0.4076 | 1.15E-11 | 1.43E-08 |
| PNMA3 | 2.0707 | 0.1956 | 1.21E-11 | 1.43E-08 |
| ELK1 | 1.4823 | 0.2850 | 1.39E-11 | 1.51E-08 |
| CD9 | -1.1858 | -0.0768 | 2.02E-11 | 2.05E-08 |
| CPS1 | 2.0698 | -0.0186 | 2.46E-11 | 2.32E-08 |
| ITGB4 | -1.8124 | -0.2237 | 2.95E-11 | 2.61E-08 |
| ITPK1 | -0.9000 | -0.1325 | 3.48E-11 | 2.85E-08 |
| PPAP2A | -1.6160 | -0.2827 | 3.70E-11 | 2.85E-08 |
| FAM98A | 0.7226 | 0.0841 | 3.82E-11 | 2.85E-08 |
| AMMECR1 | 1.2892 | -0.0505 | 4.34E-11 | 3.07E-08 |
| SORT1 | -1.1349 | -0.1108 | 8.76E-11 | 5.90E-08 |
| PGM1 | 0.8562 | 0.1214 | 9.25E-11 | 5.96E-08 |
| SFRP2 | -4.0946 | 0.3572 | 1.09E-10 | 6.36E-08 |
| SLC24A6 | 1.0261 | 0.2108 | 1.10E-10 | 6.36E-08 |
| ZNF436 | 1.0505 | 0.0807 | 1.12E-10 | 6.36E-08 |
| SCARB1 | 1.7693 | 0.2758 | 1.47E-10 | 8.02E-08 |
| TNC | -2.6291 | -0.0228 | 1.87E-10 | 9.79E-08 |
| PPAP2A | -1.4746 | -0.3588 | 1.96E-10 | 9.90E-08 |
| FAM113B | 1.3040 | 0.2737 | 2.29E-10 | 1.12E-07 |
| SOX8 | -2.1707 | -0.1509 | 2.50E-10 | 1.18E-07 |
| ARHGAP25 | 2.1140 | 0.5408 | 3.60E-10 | 1.65E-07 |
| ZMYM3 | 1.1063 | 0.1386 | 3.72E-10 | 1.65E-07 |
| DNAJC15 | -1.7541 | -0.1458 | 4.91E-10 | 2.06E-07 |
| CAMK1G | 2.2749 | 0.5714 | 4.95E-10 | 2.06E-07 |
| DDEFL1 | 1.0417 | 0.1516 | 7.17E-10 | 2.90E-07 |
| TRIM24 | 1.0100 | 0.1356 | 7.39E-10 | 2.91E-07 |
| TYRO3 | 1.7073 | 0.0478 | 1.01E-09 | 3.83E-07 |
| D4S234E | 2.8137 | 0.2914 | 1.03E-09 | 3.83E-07 |
| DKFZP761P0423 | 1.6019 | 0.3001 | 1.15E-09 | 4.17E-07 |
| TSHZ1 | 1.1663 | 0.1413 | 1.40E-09 | 4.86E-07 |
| TACC2 | -1.5373 | -0.0920 | 1.41E-09 | 4.86E-07 |
| C5ORF25 | 1.4040 | 0.1175 | 1.47E-09 | 4.94E-07 |
| LOC401089 | 2.7782 | 0.0867 | 1.99E-09 | 6.56E-07 |
| MSH6 | 0.7195 | 0.0977 | 2.54E-09 | 8.19E-07 |
| PLVAP | -2.0039 | -0.0149 | 3.08E-09 | 9.69E-07 |
| PEG3 | 2.0650 | 0.3665 | 3.19E-09 | 9.76E-07 |
| SLC9A5 | 1.0253 | 0.1107 | 3.24E-09 | 9.76E-07 |
| FNDC4 | 2.1418 | 0.3208 | 4.31E-09 | 1.27E-06 |
| LOC644311 | 1.3182 | 0.1024 | 5.52E-09 | 1.59E-06 |
| NAT9 | 0.5449 | 0.0300 | 5.80E-09 | 1.64E-06 |
| logFC = log fold change  AveExpr = average gene expression | | | | |

Table S8: Top 100 genes that were significantly (nominal p<0.05) different between medicated and non-medicated samples in OMA.

| **Genes** | **logFC** | **AveExpr** | **P.Value** |
| --- | --- | --- | --- |
| ARPM1 | -0.870 | 0.262 | 0.0001 |
| MBP | 1.503 | -0.258 | 0.0002 |
| LOC730259 | -1.327 | -0.211 | 0.0002 |
| EPB41 | 0.617 | -0.065 | 0.0004 |
| LOC201229 | -1.033 | 0.262 | 0.0005 |
| GLIPR1 | -0.768 | 0.052 | 0.0007 |
| SIP1 | -0.592 | -0.099 | 0.0008 |
| SF3B14 | -0.523 | -0.188 | 0.0010 |
| KIAA1826 | -0.492 | -0.043 | 0.0010 |
| IMPAD1 | -0.767 | -0.091 | 0.0010 |
| RGS12 | 0.629 | 0.029 | 0.0010 |
| MGC33212 | -1.353 | -0.511 | 0.0011 |
| RRAGD | 0.996 | 0.277 | 0.0015 |
| ITPR3 | 0.580 | -0.191 | 0.0015 |
| ELF2 | 0.865 | -0.007 | 0.0016 |
| MORN2 | -0.824 | -0.331 | 0.0016 |
| EID1 | -0.849 | -0.113 | 0.0019 |
| CGNL1 | -0.697 | -0.020 | 0.0019 |
| NPDC1 | 0.563 | -0.252 | 0.0023 |
| HPD | -0.889 | 0.001 | 0.0023 |
| MSI2 | -1.215 | 1.064 | 0.0023 |
| HS.436346 | 0.302 | -0.033 | 0.0025 |
| MED20 | -0.392 | -0.195 | 0.0027 |
| FASTKD3 | -0.651 | -0.131 | 0.0028 |
| PTPRA | 0.724 | -0.049 | 0.0030 |
| VBP1 | -0.621 | -0.159 | 0.0031 |
| ABCC4 | -0.515 | 0.402 | 0.0031 |
| HS.443490 | -0.536 | 0.114 | 0.0033 |
| MTCH2 | -0.528 | -0.069 | 0.0033 |
| C1ORF19 | -0.595 | -0.116 | 0.0034 |
| SULT1A3 | 0.972 | -0.266 | 0.0034 |
| SGCD | -0.949 | 0.567 | 0.0035 |
| PWWP2 | 0.538 | -0.206 | 0.0035 |
| COPS4 | -0.410 | -0.049 | 0.0035 |
| TTC26 | -0.701 | -0.010 | 0.0035 |
| LOC727948 | 0.752 | 0.122 | 0.0036 |
| LOC642897 | -0.936 | -0.360 | 0.0037 |
| ACPL2 | -0.701 | -0.075 | 0.0037 |
| SLC12A7 | 0.594 | -0.145 | 0.0038 |
| FOXC1 | 1.180 | 0.549 | 0.0040 |
| COMMD10 | -0.555 | -0.151 | 0.0042 |
| RPL37A | -0.392 | 0.243 | 0.0042 |
| DDAH1 | -0.669 | 0.162 | 0.0042 |
| ANAPC10 | -0.638 | -0.210 | 0.0043 |
| WHDC1 | 0.631 | 0.089 | 0.0045 |
| ATPAF1 | -0.520 | -0.181 | 0.0045 |
| FBXO22 | -0.872 | -0.283 | 0.0046 |
| WDR41 | -0.988 | -0.189 | 0.0048 |
| UBE2D2 | 1.098 | -0.187 | 0.0048 |
| SLC36A4 | -0.680 | 0.566 | 0.0048 |
| TWSG1 | -0.581 | 0.126 | 0.0048 |
| NME7 | -0.484 | 0.059 | 0.0049 |
| DNAJC19 | -0.653 | -0.141 | 0.0049 |
| ALS2CR4 | -0.502 | 0.181 | 0.0050 |
| HS.222909 | 0.649 | 0.807 | 0.0050 |
| IFT52 | -0.484 | 0.069 | 0.0050 |
| PREI3 | -0.598 | -0.252 | 0.0050 |
| FBXL10 | 0.346 | -0.130 | 0.0050 |
| ISCA1 | -0.414 | -0.069 | 0.0052 |
| HMG20A | -0.413 | 0.019 | 0.0052 |
| RANBP6 | -0.510 | -0.132 | 0.0052 |
| HYLS1 | -0.664 | -0.102 | 0.0053 |
| PGRMC1 | -0.673 | -0.306 | 0.0053 |
| MFAP3 | -0.635 | -0.166 | 0.0054 |
| TMEM168 | -0.662 | -0.069 | 0.0054 |
| C11ORF73 | -0.581 | -0.168 | 0.0055 |
| MRPL40 | -0.443 | -0.219 | 0.0055 |
| REPS1 | -0.570 | -0.004 | 0.0055 |
| FBXL5 | -0.514 | 0.093 | 0.0056 |
| GBP1 | -0.913 | -0.018 | 0.0056 |
| C11ORF70 | -0.938 | -0.284 | 0.0057 |
| KLHL9 | -0.363 | 0.051 | 0.0058 |
| P15RS | -0.567 | -0.181 | 0.0058 |
| USP48 | -0.643 | -0.087 | 0.0058 |
| COPS8 | -0.442 | 0.095 | 0.0058 |
| RDH14 | -0.449 | -0.224 | 0.0059 |
| PIGP | -0.803 | -0.268 | 0.0060 |
| POPDC2 | -1.276 | -0.519 | 0.0061 |
| LSM3 | -0.464 | -0.247 | 0.0062 |
| SULT1A3 | 0.725 | -0.149 | 0.0063 |
| ZNF207 | -0.310 | 0.070 | 0.0064 |
| GPR177 | -0.600 | -0.045 | 0.0064 |
| CDC2L2 | 0.880 | 0.258 | 0.0065 |
| STXBP4 | -0.896 | -0.116 | 0.0065 |
| HS.552999 | -1.135 | 0.555 | 0.0066 |
| ISOC1 | -0.703 | -0.365 | 0.0068 |
| MGC33212 | -0.666 | -0.270 | 0.0068 |
| ZNF283 | -0.466 | 0.120 | 0.0069 |
| ROR1 | -0.901 | -0.779 | 0.0070 |
| C11ORF60 | -0.298 | -0.086 | 0.0070 |
| SPATA13 | 0.973 | 0.290 | 0.0070 |
| ALG14 | -0.653 | -0.366 | 0.0071 |
| FARS2 | -0.323 | -0.338 | 0.0072 |
| DTX2 | 0.444 | 0.015 | 0.0072 |
| LOC647000 | -0.559 | 0.119 | 0.0073 |
| FAM65A | 0.365 | 0.125 | 0.0074 |
| PRTFDC1 | -0.597 | 0.241 | 0.0075 |
| NRIP2 | 0.829 | -0.047 | 0.0076 |
| PEX11B | -0.302 | -0.152 | 0.0076 |
| LOC647340 | -0.369 | -0.128 | 0.0078 |

Table S9: Top 100 genes that were significantly (nominal p<0.05)) different between medicated and non-medicated samples in SUP.

| **Genes** | **logFC** | **AveExpr** | **P.Value** |
| --- | --- | --- | --- |
| SNAP91 | 1.2635 | 0.1716 | 0.0002 |
| CDKN2D | 0.4732 | 0.0384 | 0.0002 |
| PCBP3 | 1.0727 | 0.2212 | 0.0003 |
| ZZEF1 | -0.2165 | 0.0003 | 0.0003 |
| CRLF1 | 1.4186 | 0.5551 | 0.0004 |
| RPRM | -1.0670 | -0.1407 | 0.0007 |
| PPFIBP2 | -0.5278 | 0.0594 | 0.0007 |
| SRRM1 | -0.2128 | -0.0443 | 0.0007 |
| RPL26 | -0.2551 | -0.0547 | 0.0009 |
| HS.335413 | -0.6468 | 0.0085 | 0.0010 |
| LOC728127 | -0.4024 | -0.0838 | 0.0010 |
| FAM7A3 | -0.2190 | 0.0288 | 0.0011 |
| FAM101B | 0.7431 | 0.1369 | 0.0011 |
| SEMA3B | 0.6581 | 0.2435 | 0.0012 |
| PRG2 | 0.7713 | 0.4013 | 0.0013 |
| CHST3 | 0.4224 | 0.0618 | 0.0013 |
| LOC643669 | -0.5338 | -0.0258 | 0.0014 |
| GSTO2 | -0.9255 | -0.2789 | 0.0014 |
| PDCD5 | 0.2891 | -0.1180 | 0.0014 |
| C13ORF15 | 0.8043 | 0.1487 | 0.0015 |
| SEPHS2 | -0.2596 | -0.1273 | 0.0015 |
| RTN4R | -0.6495 | 0.2035 | 0.0015 |
| KIAA1618 | -0.3853 | 0.0841 | 0.0017 |
| IFT88 | -0.5910 | -0.0482 | 0.0018 |
| NUTF2 | 0.2669 | 0.0427 | 0.0023 |
| LAS1L | -0.2153 | -0.0264 | 0.0026 |
| NFE2L1 | 0.2614 | 0.1105 | 0.0027 |
| REN | 1.2984 | 0.2955 | 0.0027 |
| VPS37D | -0.5064 | -0.1072 | 0.0028 |
| HS.190748 | 0.5312 | 0.0087 | 0.0029 |
| ADAMTS2 | 0.8593 | 0.6410 | 0.0029 |
| LOC654194 | -0.3912 | -0.0030 | 0.0030 |
| KIAA1530 | -0.3787 | -0.0577 | 0.0032 |
| TSKU | 0.7208 | 0.1479 | 0.0033 |
| C1ORF66 | -0.2429 | -0.0645 | 0.0033 |
| PKIB | -0.6415 | 0.0928 | 0.0034 |
| HS.378608 | -0.3764 | -0.0979 | 0.0035 |
| ABHD5 | 0.7681 | -0.1002 | 0.0035 |
| KL | 0.6312 | -0.0966 | 0.0035 |
| NNAT | 1.0255 | -0.3171 | 0.0036 |
| GPR162 | -0.4598 | 0.0045 | 0.0036 |
| FLCN | -0.3095 | 0.1611 | 0.0037 |
| HSD11B1 | 1.2100 | -0.1152 | 0.0037 |
| MMP25 | 0.8165 | 0.4002 | 0.0039 |
| NFX1 | -0.2133 | -0.0416 | 0.0040 |
| JMJD1B | -0.1627 | -0.0191 | 0.0041 |
| HSPC159 | 0.4796 | -0.1383 | 0.0041 |
| DHRS12 | -0.2596 | -0.0075 | 0.0042 |
| ATP6V1E2 | -0.3478 | -0.0776 | 0.0042 |
| TIMP3 | 0.8717 | 0.4482 | 0.0042 |
| KCTD14 | -0.6927 | 0.0211 | 0.0042 |
| CAB39L | 1.0409 | 0.2700 | 0.0043 |
| AADACL4 | 0.7432 | 0.2876 | 0.0043 |
| PPM1B | -0.5207 | -0.0908 | 0.0043 |
| NR2F1 | -0.6398 | 0.1671 | 0.0043 |
| FUBP1 | -0.3561 | -0.1232 | 0.0045 |
| RORA | -0.3655 | -0.0705 | 0.0046 |
| APOBEC3F | -0.5007 | -0.0056 | 0.0047 |
| PEX5 | -0.2076 | -0.0440 | 0.0048 |
| MT1G | -1.3907 | -0.5160 | 0.0048 |
| RUFY1 | -0.2026 | 0.0237 | 0.0048 |
| C6ORF106 | -0.2159 | -0.0405 | 0.0048 |
| SIDT2 | -0.2768 | -0.0038 | 0.0048 |
| KIAA0174 | -0.1145 | 0.0127 | 0.0049 |
| CCDC24 | -0.4689 | -0.1182 | 0.0051 |
| PLCB1 | -0.1759 | -0.0353 | 0.0052 |
| LOC653344 | -0.5234 | -0.2002 | 0.0053 |
| HS.374023 | 0.5180 | -0.0143 | 0.0054 |
| CRYL1 | -0.2780 | 0.0022 | 0.0055 |
| FXYD1 | 0.8015 | -0.0492 | 0.0055 |
| AFF1 | -0.1731 | -0.0074 | 0.0055 |
| FBXO42 | -0.2123 | -0.0004 | 0.0055 |
| AP1GBP1 | -0.3074 | -0.0229 | 0.0059 |
| IGFBP4 | 0.3401 | 0.1843 | 0.0060 |
| RIMBP2 | -0.6686 | 0.2583 | 0.0061 |
| APOO | 0.3659 | -0.0377 | 0.0061 |
| ZCCHC8 | -0.1943 | -0.0121 | 0.0062 |
| WDR91 | -0.3362 | -0.0780 | 0.0064 |
| S100A8 | 1.3318 | 0.4285 | 0.0064 |
| KIAA1958 | -0.2865 | -0.1634 | 0.0067 |
| INPP4B | 0.2047 | 0.0299 | 0.0068 |
| ZNF250 | -0.3499 | -0.0755 | 0.0068 |
| ING1 | 0.3877 | 0.0921 | 0.0069 |
| TAPBPL | -0.3419 | 0.0967 | 0.0071 |
| WDR6 | -0.2042 | -0.0347 | 0.0071 |
| REXO4 | -0.1937 | -0.0296 | 0.0072 |
| CCDC113 | -0.9203 | -0.2561 | 0.0072 |
| ARL4A | -0.5666 | 0.0030 | 0.0073 |
| MRPL39 | -0.1036 | -0.0126 | 0.0074 |
| MGC5139 | -0.1482 | -0.0411 | 0.0074 |
| CDK5RAP1 | -0.1530 | -0.0553 | 0.0075 |
| ANPEP | 0.6709 | 0.1898 | 0.0075 |
| TRRAP | -0.1489 | 0.0095 | 0.0075 |
| COPS8 | 0.2886 | 0.0145 | 0.0076 |
| DNAJC4 | -0.3343 | 0.0187 | 0.0076 |
| LOC440731 | 0.4735 | -0.1523 | 0.0076 |
| TOP1 | -0.3852 | -0.1747 | 0.0076 |
| CGNL1 | -0.5455 | 0.0527 | 0.0077 |
| AXIN2 | -0.4013 | 0.2766 | 0.0080 |
| UCHL1 | 0.6645 | 0.3525 | 0.0082 |
| logFC = log fold change  AveExpr = average gene expression | | | |

Table S10: Top 100 genes that were significantly (nominal p<0.05)) different between medicated and non-medicated samples in DIE.

| **Genes** | **logFC** | **AveExpr** | **P.Value** |
| --- | --- | --- | --- |
| COPS8 | 0.27622 | 0.08750 | 0.00027 |
| PHF19 | 0.47529 | 0.01991 | 0.00031 |
| MKRN1 | -0.19767 | -0.10170 | 0.00125 |
| LOC653527 | 1.22324 | -0.16880 | 0.00137 |
| FLJ20581 | -0.16978 | 0.01834 | 0.00142 |
| ZNF69 | -0.47731 | -0.18187 | 0.00147 |
| LGALS1 | 0.21096 | 0.01836 | 0.00151 |
| AKAP8L | -0.29748 | -0.07099 | 0.00155 |
| LOC645317 | 0.59919 | -0.06417 | 0.00160 |
| YWHAG | 0.31277 | 0.06380 | 0.00166 |
| PHLDB3 | -0.44003 | 0.08938 | 0.00167 |
| C20ORF20 | -0.16853 | -0.12320 | 0.00174 |
| ALS2CR13 | -0.34637 | -0.33593 | 0.00180 |
| HS.374023 | 0.64622 | 0.08191 | 0.00184 |
| PNPLA8 | 0.38973 | 0.38488 | 0.00192 |
| STXBP1 | 0.42384 | 0.05235 | 0.00204 |
| EGLN1 | 0.38500 | 0.01007 | 0.00228 |
| LONRF2 | 0.70144 | 0.40391 | 0.00236 |
| ACVR2B | -0.32537 | -0.15757 | 0.00243 |
| KIAA1530 | -0.29217 | 0.02472 | 0.00252 |
| CPS1 | 0.77142 | -0.38318 | 0.00257 |
| HS.492187 | -0.25329 | -0.06780 | 0.00270 |
| LOC221710 | 0.41652 | 0.21034 | 0.00339 |
| TWIST2 | 0.73906 | 0.43476 | 0.00353 |
| FADS1 | 0.32592 | -0.02632 | 0.00358 |
| WDR18 | -0.24916 | -0.22762 | 0.00369 |
| RUNDC2C | -0.48256 | -0.04957 | 0.00371 |
| CEL | -0.72921 | -0.72833 | 0.00372 |
| ANKZF1 | -0.30246 | -0.25864 | 0.00375 |
| PLCL1 | 1.02559 | 0.04532 | 0.00377 |
| LOC493754 | 0.30697 | 0.40447 | 0.00380 |
| RSRC1 | 0.38302 | 0.37499 | 0.00385 |
| IFT172 | -0.28500 | -0.27321 | 0.00402 |
| AHCTF1 | 0.47617 | 0.39726 | 0.00406 |
| ANXA11 | -0.22429 | 0.07251 | 0.00415 |
| LOC646139 | -0.35731 | -0.03774 | 0.00424 |
| SON | 0.30781 | 0.15113 | 0.00425 |
| C1ORF152 | -0.59819 | -0.24111 | 0.00430 |
| SMAD9 | -0.70720 | -0.15185 | 0.00444 |
| EFNA1 | -0.41371 | -0.36548 | 0.00447 |
| PARC | -0.25315 | -0.10866 | 0.00448 |
| EIF2C3 | 0.28681 | 0.00869 | 0.00455 |
| LYAR | 0.36076 | 0.33315 | 0.00455 |
| PPP1R13B | -0.29741 | -0.38734 | 0.00472 |
| GUSBL2 | -0.26444 | -0.17867 | 0.00478 |
| BTG3 | 0.30119 | 0.37372 | 0.00483 |
| C14ORF149 | 0.46798 | -0.03636 | 0.00489 |
| LSS | 0.22935 | -0.12192 | 0.00491 |
| ERICH1 | 0.22546 | 0.03698 | 0.00537 |
| SLC16A5 | -0.36248 | -0.24232 | 0.00543 |
| HS.562214 | -0.13267 | 0.03360 | 0.00566 |
| VARS2 | -0.24748 | -0.33636 | 0.00645 |
| MSL3L1 | -0.39283 | 0.02895 | 0.00669 |
| C5AR1 | 0.79105 | 0.75786 | 0.00670 |
| RPS26L1 | -0.59473 | 0.01938 | 0.00676 |
| EXOC7 | -0.16572 | -0.09733 | 0.00697 |
| PPFIBP2 | -0.46224 | -0.42004 | 0.00707 |
| COMMD5 | -0.26681 | -0.07383 | 0.00717 |
| FAM49A | 0.39763 | 0.46810 | 0.00730 |
| C17ORF70 | -0.22587 | -0.22417 | 0.00747 |
| VENTX | 0.54448 | 0.32623 | 0.00764 |
| TRPC4 | 0.34208 | 0.13762 | 0.00765 |
| SPTLC1 | 0.40678 | 0.09288 | 0.00766 |
| CXXC1 | -0.15323 | -0.17449 | 0.00772 |
| KIAA0652 | -0.21562 | -0.16939 | 0.00780 |
| SFRP2 | -1.06653 | 0.95655 | 0.00781 |
| NEK8 | -0.47309 | -0.62473 | 0.00805 |
| BMF | -0.78777 | -0.96755 | 0.00806 |
| ENPEP | 0.71817 | 0.06831 | 0.00822 |
| SLC2A4RG | 0.25009 | -0.11862 | 0.00838 |
| CLEC16A | -0.14901 | -0.22870 | 0.00840 |
| LOH3CR2A | 0.93218 | 1.11357 | 0.00850 |
| FKBP5 | 0.62046 | 0.67081 | 0.00893 |
| FOXO1 | 0.40465 | 0.09852 | 0.00903 |
| LOC441066 | -0.57279 | -0.23280 | 0.00905 |
| TBCD | -0.18464 | -0.20773 | 0.00921 |
| USP48 | 0.27775 | 0.09802 | 0.00923 |
| CDC2L2 | -0.40143 | 0.07225 | 0.00931 |
| USMG5 | 0.83721 | 0.27432 | 0.00939 |
| SNAPC4 | -0.16939 | -0.28008 | 0.00947 |
| LOC654260 | -0.28753 | -0.38107 | 0.00955 |
| MAMDC2 | 0.61427 | 0.76513 | 0.00964 |
| CITED2 | 0.53818 | 0.34238 | 0.00968 |
| PTCH1 | -0.79367 | -0.50491 | 0.00985 |
| FAM134C | -0.21531 | -0.11078 | 0.00988 |
| HS.473255 | -0.26119 | -0.05294 | 0.01010 |
| TDG | -0.23242 | 0.01918 | 0.01016 |
| PID1 | 0.56174 | 0.46686 | 0.01017 |
| CDRT4 | -0.34898 | -0.25506 | 0.01025 |
| ABCC5 | -0.29004 | -0.17085 | 0.01042 |
| UBAP2 | -0.13046 | -0.16035 | 0.01046 |
| HS.535044 | -0.50913 | -0.05790 | 0.01050 |
| FLJ46380 | -0.42670 | -0.11483 | 0.01062 |
| LRDD | -0.25084 | -0.23048 | 0.01066 |
| RAD9A | -0.27984 | -0.32701 | 0.01083 |
| SENP5 | 0.32000 | 0.36090 | 0.01084 |
| HYDIN | 0.13208 | 0.02763 | 0.01087 |
| HS.171485 | 0.55921 | 0.61066 | 0.01094 |
| SCCPDH | -0.26839 | -0.04847 | 0.01110 |
| ITGA3 | -0.35334 | -0.00643 | 0.01122 |
| logFC = log fold change  AveExpr = average gene expression | | | |
